# A spatially resolved genomic–molecular atlas of human white-matter microstructure

**DOI:** 10.64898/2026.07.06.26357381

**Published:** 2026-07-09

**Authors:** Alec Reinhardt, Zhiwen Jiang, Tengfei Li, Borui Tang, Xinjie Qian, Shuai Huang, Joseph G. Ibrahim, Yun Li, Bingxin Zhao, Patrick Sullivan, Jason Stein, Hongtu Zhu

**Affiliations:** 1 Department of Biostatistics, University of North Carolina at Chapel Hill; 2 Biomedical Research Imaging Center, UNC School of Medicine; 3 Department of Radiology, UNC School of Medicine; 4 Department of Genetics, University of North Carolina at Chapel Hill; 5 Department of Statistics and Data Science, The Wharton School, University of Pennsylvania; 6 Department of Psychiatry, University of North Carolina at Chapel Hill

## Abstract

Human white matter has been linked to inherited variation, circulating molecular state and brain disease, but these layers have rarely been mapped onto the same tract anatomy. Here we measured genetic effects along 6,090 atlas-aligned fiber pathways sampled at 609,000 locations in 72,185 UK Biobank participants, and integrated proteomic and metabolomic profiles within the same anatomical frame. Genetic effects were not whole-tract properties: each locus formed a spatial footprint along fiber trajectories, ranging from single locations to broad multi-tract patterns and reflecting regional polygenicity rather than tract heritability. This map identified 258, 186 and 298 previously unreported loci for fractional anisotropy, mean diffusivity and axial diffusivity; spatial patterns replicated in adults and 157 of 315 FA loci replicated in adolescence in ABCD. Mendelian randomization linked localized genetic effects to neurodegenerative and psychiatric traits, with Alzheimer’s disease showing directional effects across 12 of 17 tracts. Multi-omic analyses identified 97 proteomic and 161 metabolomic associations, with the broadest signals from lipid metabolites including linoleic acid and phosphatidylcholines. The strongest lipid-metabolite and genetic signals converged in the corpus callosum, placing inherited variation, disease risk and systemic lipid metabolism on the same localized tract segments.

## Introduction

White-matter tracts connect cortical and subcortical systems and support communication across the human brain [1,2]. Their microstructure is central to cognition, behavior and brain health, and is altered in many neuropsychiatric and neurodegenerative disorders [3,4]. Diffusion MRI measures this microstructure in vivo through indices such as fractional anisotropy, mean diffusivity and axial diffusivity, which capture complementary features of axonal coherence, myelination and tissue organization [5-9]. Twin and family studies have long shown that these measures are substantially heritable [10-12], and recent large-scale genome-wide association studies have revealed a highly polygenic architecture with genetic overlap with cognitive and psychiatric traits [13-15].

Despite these advances, most genetic studies of white matter still reduce the diffusion signal before testing. Tract-averaged measures, tract-level functional principal components [13], structural connectome connectivity phenotypes [16,17], and global latent representations of whole-brain diffusion maps [18] each summarize white matter at a scale broader than a specific position along a tract. These approaches have expanded the field, but they cannot determine where within an individual tract a genetic association is strongest. It therefore remains unclear whether common variants act broadly across a tract or concentrate at localized tract segments that are obscured by aggregate phenotypes.

A second gap is molecular. GWAS can identify loci and implicate genes or biological pathways, but it does not directly show how inherited variation relates to measured molecular state. Circulating proteins and metabolites provide complementary readouts of systemic biology, including lipid metabolism, inflammation and extracellular signaling. Yet these molecular phenotypes have not been integrated with white-matter genetics at the same anatomical resolution [19]. Previous multi-omics analyses have relied mainly on annotations inferred from GWAS results, such as gene sets, pathway enrichment or predicted protein networks, rather than measured proteins and metabolites mapped onto the same white-matter anatomy [18]. Whether inherited variation, molecular state and disease risk localize to the same tract segments has therefore remained unresolved.

Here we address these gaps by building a molecular-genetic atlas of human white matter. We combined a connectome-based spatial statistics framework that samples diffusion signals along atlas-aligned fiber trajectories [25] with a representation-learning strategy for efficient genome-wide testing of high-dimensional imaging phenotypes [26]. Using diffusion MRI from 56,313 UK Biobank participants for discovery and an independent UK Biobank cohort for validation, we mapped genetic effects along 6,090 fiber pathways sampled at 609,000 locations and integrated proteomic and metabolomic phenotypes within the same white-matter anatomy, and we tested developmental generalization in the Adolescent Brain Cognitive Development study. This location-level map showed that genetic effects are not simply properties of whole tracts but form spatial footprints along fiber trajectories, ranging from single locations to broad multi-tract patterns and revealing regions where genetic signal concentrates beyond what tract-level heritability would predict. Mendelian randomization and colocalization linked these localized effects to neuropsychiatric and neurodegenerative disease, including Alzheimer's disease. Finally, proteomic and metabolomic analyses showed that lipid metabolites, including linoleic acid and phosphatidylcholines, overlapped genetic signals most strongly along callosal pathways. Together, these findings place inherited disease risk and systemic lipid metabolism in the same white-matter anatomy, providing a spatial framework for studying the molecular organization of white-matter microstructure.

## Results

### Representation learning and quality control of location-level diffusion metrics

To enable fine-grained genetic analysis of white-matter microstructure at biobank scale while preserving anatomical interpretability, we used the connectome-based spatial statistics (CBSS) framework [25]. CBSS encodes diffusion MRI signals along atlas-aligned fiber trajectories rather than isolated voxels, maintaining tract topology and functional network organization (Fig. S1). The white-matter skeleton was reconstructed into 6,090 fiber bundles, each sampled at 100 along-tract nodes, yielding 609,000 ordered fiber-grid locations. To reduce redundancy and ensure computational efficiency for genome-wide testing, these locations were hierarchically aggregated into 430 anatomically coherent clusters (Table S1), constrained by functional network compartments and local neighborhoods. This preserves biological organization while allowing full reconstruction of location-level signals.

Within each cluster, we applied functional principal component analysis (fPCA) to derive low-dimensional representations (LDRs) [26]. Conceptually, fPCA extracts smooth spatial modes of microstructural variation along fiber trajectories, identifying coherent patterns rather than voxelwise noise. Graphically, each LDR can be viewed as a compact “basis function” describing a canonical spatial motif (e.g., a gradient or focal change), and each participant’s tract profile is expressed as a weighted combination of these motifs. Dimensionality was selected to preserve 80–90% of variance per cluster, yielding on average 20.6 LDRs (8,868 total phenotypes). Reconstruction fidelity was high (correlation = 0.85–0.95; Fig. S2c), and leading components showed anatomically smooth, biologically plausible patterns (Fig. S3a,c). Wild-bootstrap null simulations demonstrated well-calibrated type-I error across tracts (Fig. S3b), supporting the validity of these representations for downstream inference.

### GWAS and heritability of white matter microstructure

**Figure 2.**
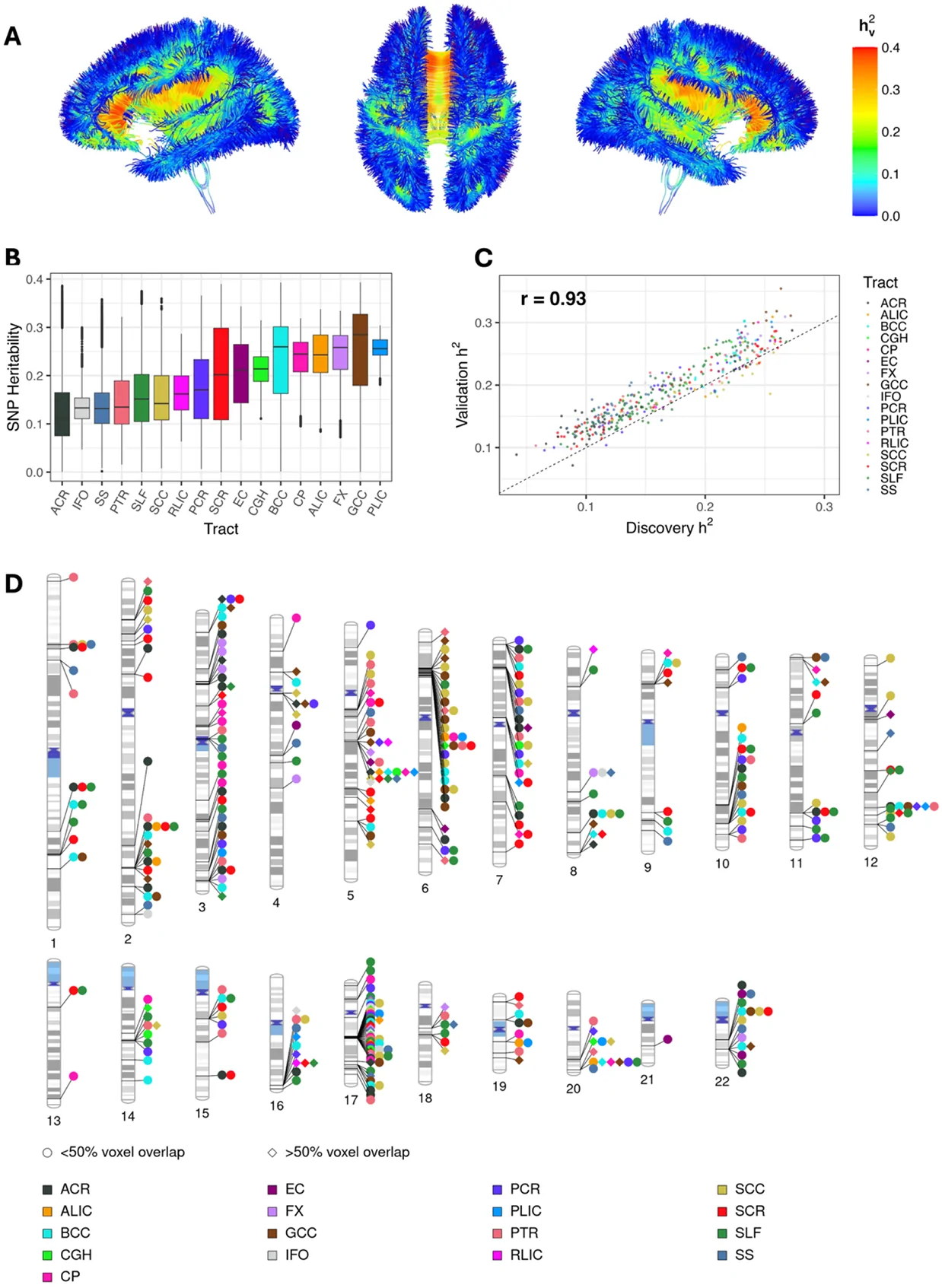
(A) Location-wise SNP heritability estimates of FA across 6,090 fibers. Axial, sagittal, and coronal slices are shown. (B) Distributions of SNP heritability estimates stratified by white matter tract (17 distinct ROIs). (C) Concordance in SNP heritability estimates between discovery (UKB Phases 1-6) and validation (UKB Phase 7) cohorts. Points represent mean SNP heritability per fiber cluster (430 total), and colors represent assigned tracts. (D) Ideogram of genomic regions influencing FA parameters (P < 9.12e-12) for the 407 tract-level genetic loci which were spatially replicated in the validation cohort. Colors represent the 17 assigned tracts, and shapes indicate whether the proportion of significant voxels for loci between discovery and validation cohorts was less than or greater than 50%.

Using the UK Biobank discovery cohort (Phase 1–6; n = 56,313, white European ancestry), we performed GWAS on 8,868 low-dimensional FA representations spanning 430 fiber clusters (609,000 locations). Projecting summary statistics back into image space revealed extensive genetic influence on white-matter microstructure: after correction for the effective number of voxel-level tests (P < 9.12 × 10^−6^), 90.6% of locations were significantly heritable in FA, with h_v_^2^ ranging from 0 to 39.3% (mean = 16.4%) [Fig. 2a, Fig. S4, Tab. S2]. Aggregated estimates confirmed robust heritability across all 17 major tracts (mean = 19.3%) and 66 functional network pairs (mean = 17.3%) [Fig. 2b, Table S2]. We compared the heritability estimates for FA with the MD and AD modalities, and found largely consistent spatial profiles (see Supplementary Details).

Heritability patterns exhibited clear anatomical organization. The posterior limb of the internal capsule (PLIC) showed the highest and most uniform heritability (mean = 25.6%, SD = 2.7%), consistent with strong genetic regulation of tightly packed projection pathways. In contrast, association and callosal systems such as the superior corona radiata (SCR) (mean = 20.2%, SD = 10.0%) and genu of the corpus callosum (GCC) (mean = 25.1%, SD = 9.5%) displayed greater within-tract variability, reflecting more heterogeneous microstructural organization. These spatial patterns were highly reproducible in the UKB Phase 6 cohort (n = 14,965; r = 0.72 location-wise; r = 0.93 cluster-wise) [Fig. 2c; Table S2].

Applying both genome-wide significance (P < 5 × 10^−8^; voxel-adjusted P < 9.12 × 10^−12^) and empirical spatial screening identified 1,182 tract-level loci, which consolidated into 315 distinct loci; parallel analyses of MD and AD identified 238 and 345 distinct loci, respectively. The FA loci spatial footprints ranged from focal (single-location) to highly distributed (up to 185,624 locations; median = 118), and nearly half (47.6%) influenced more than two tracts, indicating a mixture of pathway-specific and cross-system genetic effects. Replication in Phase 6 was strong: 311 of 315 (98.7%) FA loci showed directional concordance, effect sizes were highly correlated (slope = 0.68, SE = 0.05, P < 2 × 10^−16^), and 44% demonstrated >50% spatial overlap [Fig. 2d]; directional replication was similarly robust for MD (233/238, 97.9%) and AD (339/345, 98.3%), with 40% and 38% of loci demonstrating >50% spatial overlap, respectively. Compared with the largest prior GWAS of white-matter microstructure [13], we replicated 57, 52, and 46 previously reported FA, MD, and AD loci and discovered 258, 186, and 298 novel loci, respectively, 115 of the novel FA loci also showing >50% spatial concordance.

GWAS Catalog lookup indicated that 88 of 315 FA loci (27.9%) had been previously linked to FA or other white-matter phenotypes, while the remaining loci showed broad pleiotropy: 293 loci (93.0%) overlapped regions associated with neuropsychiatric or cognitive traits (395 traits total), including educational attainment (120 loci), schizophrenia (27), AD (20), major depressive disorder (18), bipolar disorder (9), and Parkinson’s disease (7). More than 70% also overlapped loci for non-neurological traits—such as bone density (83.4%), cardiac function (81.4%), and refractive error (71.2%)—highlighting the cross-organ pleiotropic architecture underlying white-matter biology.

Location-level spatial atlases clarified this architecture. Many loci produced focal, tract-specific signatures concentrated in projection pathways (e.g., PLIC, forceps major), consistent with variants affecting axon guidance, axon caliber, and myelination programs that follow fixed developmental trajectories — exemplified by the 18p11.21 locus (rs2306811), which showed a spatially concentrated footprint restricted to the body and genu of the corpus callosum and superior corona radiata. Others showed broad, distributed signatures spanning multiple association fibers and callosal tracts, implicating pathways related to oligodendrocyte function, axonal maintenance, extracellular matrix remodeling, and neuroimmune signaling [Fig. S5]; the 12q23.3 locus (rs12146713) illustrates this pattern strikingly, with associations spanning 12 FA tracts — including the corpus callosum, internal capsule, corona radiata, and superior longitudinal fasciculus — and 71.8% voxel-level spatial overlap in the replication cohort. Hotspot maps converged on the corpus callosum, corticospinal tract, and fronto-occipital fasciculus, identifying these as genetic hubs of white-matter organization, consistent with pronounced locus enrichment in compact projection and limbic tracts relative to their fiber-skeleton footprint (ALIC: OR = 54; FX: OR = 42; PLIC: OR = 37). Notably, these hotspot patterns were not explained by tract-level heritability alone, indicating that the spatial concentration of associations reflects regional polygenicity and effect-size distribution rather than overall h^2^ magnitude. Together, these results reveal a hierarchical genetic architecture in which projection systems are governed by tightly localized programs, whereas association and callosal networks are shaped by broader, multi-tract influences.

### Validation on ABCD

To assess the generalizability of the identified loci, we performed external validation in the Adolescent Brain Cognitive Development (ABCD) cohort (n = 7500). Given the reduced sample size relative to the UK Biobank, replication was defined based on consistency of spatial signal rather than genome-wide significance, using a permissive threshold (p < 1×10^−2^) combined with spatial overlap criteria. Across the 315 FA loci identified in discovery, 157 (49.8%) demonstrated evidence of replication in ABCD, defined as loci exhibiting >50% spatial overlap between discovery and validation voxel-level association maps. Among these, 77 (24.4%) loci showed consistent replication in both the UKB Phase 7 validation cohort and ABCD, indicating robust cross-cohort reproducibility. For MD, 87 of 186 loci (46.8%) met the ABCD replication criterion, including 42 loci (22.6%) that replicated in both UKB Phase 7 and ABCD. Similarly, 146 of 298 AD loci (49.0%) exhibited evidence of replication in ABCD, of which 71 (23.8%) were consistently replicated across both validation cohorts.

Replicated loci in ABCD exhibited similar anatomical patterns broadly consistent with those observed in the discovery cohort across all three diffusion modalities. For FA and AD, replicated associations were enriched within projection and callosal pathways, particularly the posterior thalamic radiation (PTR), sagittal stratum (SS), body of the corpus callosum (BCC), superior corona radiata (SCR), and fornix (FX), suggesting that reproducible genetic effects preferentially localize to core white-matter systems. MD-associated loci showed a similar but somewhat greater concentration within association pathways, including the superior longitudinal fasciculus (SLF), alongside callosal and projection fibers. Notably, loci associated with neurodegenerative traits showed preferential replication within the superior corona radiata (SCR), which accounted for a substantial proportion of tract-specific signals within this group (~37%), whereas loci linked to psychiatric traits demonstrated more diffuse patterns spanning a broad set of white-matter tracts. Despite differences in cohort composition and developmental stage, the spatial organization of genetic effects remained largely preserved across modalities, supporting the robustness and biological relevance of the identified white-matter associations.

### Links between white matter microstructure and diseases

**Figure 3.**
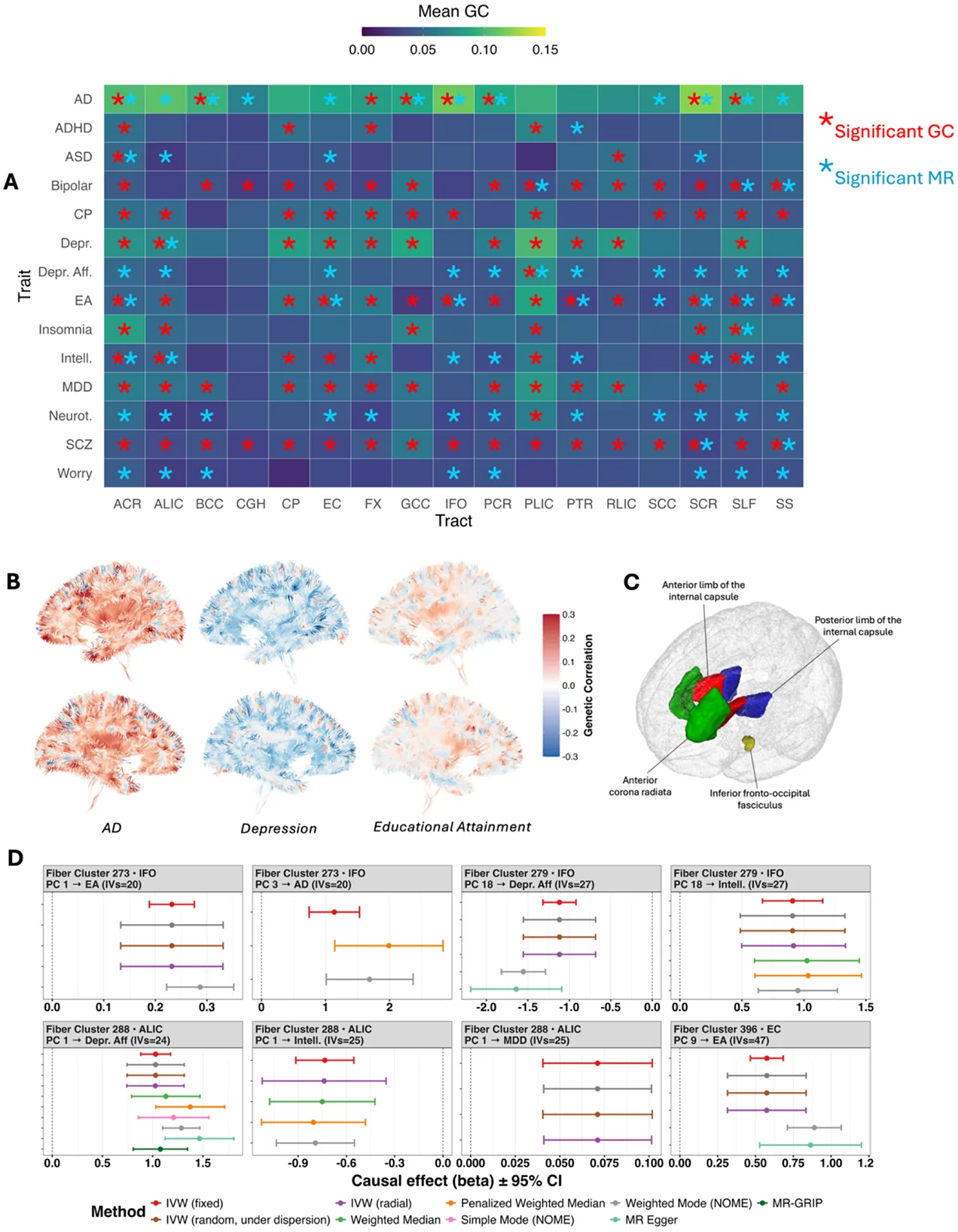
(A) Heatmap of genetic correlation (GC) and Mendelian randomization (MR) results for 17 white-matter tracts and 14 brain-related traits. Heatmap color represents the mean absolute GC within each tract. Red stars indicate significant GC after FDR correction based on tract-level p-values obtained using the Cauchy combination test. Blue stars indicate significant causal effects from two-sample MR, defined as FDR-significant IVW estimates with support from at least three additional MR methods showing concordant effect directions across LDRs within the tract.

We quantified shared polygenic architecture between FA and multiple neuropsychiatric, neurodegenerative, and cognitive traits using linkage disequilibrium score regression (LDSC). Location-wise genetic correlations (r_9_) revealed distributed, network-structured patterns across the white-matter skeleton (Fig. 3a, c). Schizophrenia, educational attainment, and bipolar disorder showed the broadest signatures, each with significant correlations in ~1% of locations after correction (P < 9.12 × 10^−6^; Tab. S3). Averaged across the brain, AD exhibited the strongest positive genome-wide correlation (weighted mean r_9_ = 0.086), while MDD showed the strongest negative correlation (weighted mean r_9_ = −0.052). Tract-level aggregation identified 112 significant tract-trait pairs (FDR < 0.05) [Fig. 3a], with the most prominent network-level associations mapping onto the superior corona radiata and inferior fronto-occipital fasciculus for AD, and the posterior limb of the internal capsule for depression (Tab. S3). Directional agreement within tracts (76.7%) indicated coherent genetic effects along shared network pathways.

To test whether these correlations reflected shared causal signals, we performed Bayesian colocalization at 151 replicated FA loci. Applying stringent thresholds (PP.H4 ≥ 0.8 and PP.H4 > PP.H3), 47 loci colocalized with at least one external phenotype across 17 traits (Table S4). Notably, 35 were novel relative to prior white-matter GWAS, demonstrating that newly discovered FA loci intersect established neuropsychiatric risk regions at the level of shared causal variants. Colocalization spanned both brain-specific pathways (e.g., schizophrenia: 8 loci; educational attainment: 7 loci) and systemic physiology (e.g., glomerular filtration rate: 9; type 2 diabetes: 7). For prioritized loci, aligned association peaks across FA and external traits supported shared local genetic drivers (Fig. S6-S10).

We next examined directional effects using two-sample Mendelian randomization. Under a conservative multi-estimator framework requiring FDR-corrected IVW significance and ≥4 concordant MR methods, 71 tract-trait pairs showed robust directional associations (Fig. 3c). These effects were strongly network-dependent: AD exhibited directional influence in 12 of 17 tracts, while neuroticism and depressed affect showed widespread signals (13 and 12 tracts, respectively). Educational attainment and intelligence showed intermediate patterns (9 and 8 tracts). In contrast, schizophrenia—despite broad spatial genetic correlations—showed directional evidence in only 3 tracts, suggesting that its white-matter associations primarily reflect pleiotropy rather than causal influence.

Across traits, directional signals concentrated in major association pathways, including the superior longitudinal fasciculus, superior corona radiata, sagittal stratum, and anterior limb of the internal capsule (Fig. 3d)—networks repeatedly implicated in AD, affective disorders, and large-scale cognitive integration. Significant pairs were supported by a mean of 7–9 MR estimators, underscoring analytical robustness (Fig. 3e).

Together, these results show that white-matter microstructure shares extensive network-level polygenic architecture with brain-related traits, yet exhibits trait-specific causal pathways: strong for AD and internalizing phenotypes, intermediate for cognitive traits, and limited for schizophrenia. This pattern highlights a hierarchical organization in which association-network microstructure serves as a key substrate linking genetic risk to neuropsychiatric and neurodegenerative phenotypes.

### Multi-omics association atlases

We next examined molecular correlates of white-matter microstructure by performing location-level phenome-wide association analyses (PheWAS) between FA and three circulating molecular modalities—proteomics, metabolomics, and clinical laboratory biomarkers—measured from peripheral venous blood in UK Biobank Phases 1–6. For each modality, location-wise association statistics were obtained from linear models adjusted for the same demographic and genetic covariates used in the GWAS, with sample sizes of 5,614 (proteomics), 26,412 (metabolomics), and 18,689 (clinical biomarkers). Multiplicity correction accounted for the effective number of independent locations and omics features, and spatial screening was performed using a wild-bootstrap null model. This analysis identified 97 proteomic and 161 metabolomic features with significant location-level associations with FA, whereas no clinical biomarkers passed both statistical and spatial criteria.

**Figure 4.**
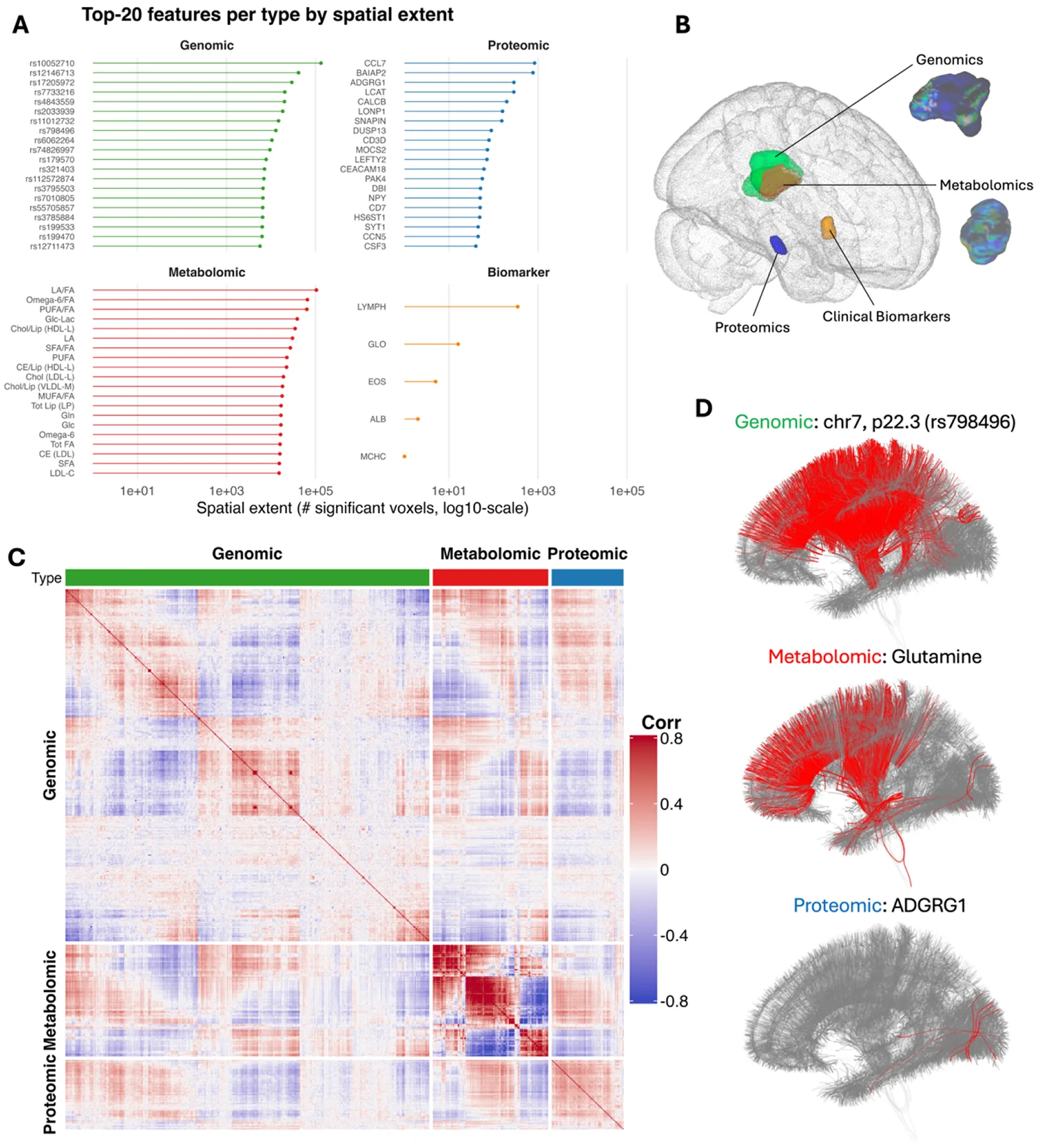
Selected top features per omics type ranked by number of significantly associated voxels. For genomic features, independent variants were identified with significance threshold P<1e-8/5479.48=9.12e-12 followed by LD clumping. For other omics types, a screening threshold of P < 0.05/(N_eff_ 5479.48) was used, based on the effective number of features within that omics type. (B) Brain atlas indicating sub-tract regions with significant associations in >1% of omics features of each type. Colors indicate omics type. Zoomed topologies are displayed for genomic and metabolomic features, indicating the variation in proportions of significant features across voxels. (C) Heatmap displaying correlations in voxel-wise effects estimates for genomic, metabolomic, and proteomic features identified to be significantly associated with FA parameters. (D) Fiber-level atlases showing significance for selected multi-omics features. Red lines indicate that >50% of voxels within that fiber were found to be significant after applying effective number multiplicity correction. Gray lines represent fibers with <50% of voxels with significant associations.

Metabolomic features showed the broadest and most anatomically coherent associations, with extensive clusters of significant voxels across major projection and callosal pathways—most prominently the corpus callosum (Fig. 4a,b). The strongest associations involved linoleic acid, phosphatidylcholines, and carnitine-related metabolites, whose regional effects concentrated in commissural and corticospinal tracts, consistent with known roles in lipid metabolism, myelin structure, and axonal energy support. Notably, regions showing significant metabolomic associations displayed high spatial concordance with genomic association hotspots (Dice = 0.67), suggesting that a shared set of anatomical hubs integrates both genetic and metabolic influences on white-matter microstructure. In contrast, proteomic associations were sparser and more spatially restricted, and clinical biomarkers showed no significant spatial patterns.

To assess cross-omic structure, we computed pairwise Pearson correlations of location-wise Z-score maps (Fig. 4c). Effect-map clustering occurred strongly by omics type, with metabolomic features exhibiting the highest within-type concordance (median ∣r∣ = 0.6). Strikingly, we observed numerous feature pairs with minimal phenotypic correlation (∣r∣ < 0.1) but highly concordant spatial association patterns. For example, circulating calcitonin gene-related peptide beta (CALCB) showed weak phenotypic correlations with LCAT (r = −0.008) and BID (r = −0.036), yet their FA effect maps were strongly aligned (r = 0.51 and 0.58). Similarly, omega-6 fatty acid percentage and glutamine were only weakly phenotypically correlated (r = 0.058) but demonstrated substantial spatial concordance (r = 0.78). Conversely, some phenotypically correlated features showed little overlap in their FA effects. Together, these findings show that systemically distinct molecular features can converge upon shared microstructural substrates, producing similar white-matter signatures despite minimal covariance at the circulating level (Fig. S11).

Finally, we quantified the overlap of statistically significant locations using Dice scores after FDR correction, identifying feature pairs with >0.6 spatial overlap. Several of the strongest overlaps occurred across omics layers—for example, between metabolite–gene or metabolite–variant pairs—highlighting multi-omics convergence on specific white-matter pathways (Fig. 4d). These results demonstrate that genetically and metabolically driven processes converge on the same anatomical systems, suggesting shared biological mechanisms linking systemic physiology, molecular signaling, and white-matter microstructure.

### Biological Enrichment Analysis

To place the location-level genetic and omics associations into a broader biological framework, we performed multi-layered enrichment analyses. From our GWAS results, we identified tracts with significantly enriched signals and compared these tract-level profiles across FA, MD, and AD. Using both locus-based enrichment (Fisher’s exact test) and size-normalized enrichment relative to tract-specific fiber counts, we observed consistent overrepresentation of signals in compact projection and limbic pathways, including the anterior and posterior limbs of the internal capsule (ALIC: OR = 40-56; PLIC: OR = 31-44), the fornix (OR = 27-42), the external capsule (OR = 13-35), and the cingulum (hippocampal; OR = 22-37), indicating a shared anatomical substrate of genetic effects across diffusion modalities. Despite this overall concordance, notable modality-specific differences emerged. FA-associated loci showed stronger enrichment in the cerebral peduncle (FA: OR = 13.1 versus MD: OR = 9.7, AD: OR = 9.8) and external capsule (FA: OR = 35.4 versus MD: OR = 23.0, AD: OR = 13.1). In contrast, MD showed markedly greater enrichment in the body of the corpus callosum (MD: OR = 9.0 versus FA: OR = 4.7, AD: OR = 6.1), and the posterior thalamic radiation was entirely absent from MD-associated loci (0 loci; FA: OR = 2.6, AD: OR = 2.4), suggesting differential sensitivity of diffusivity metrics to callosal and thalamic projection microstructure. Cross-modality comparison of enrichment profiles revealed high concordance (FA–MD: r = 0.96, FA–AD: r = 0.96, MD–AD: r = 0.98), supporting largely shared but non-identical genetic architectures across modalities.

**Figure 5.**
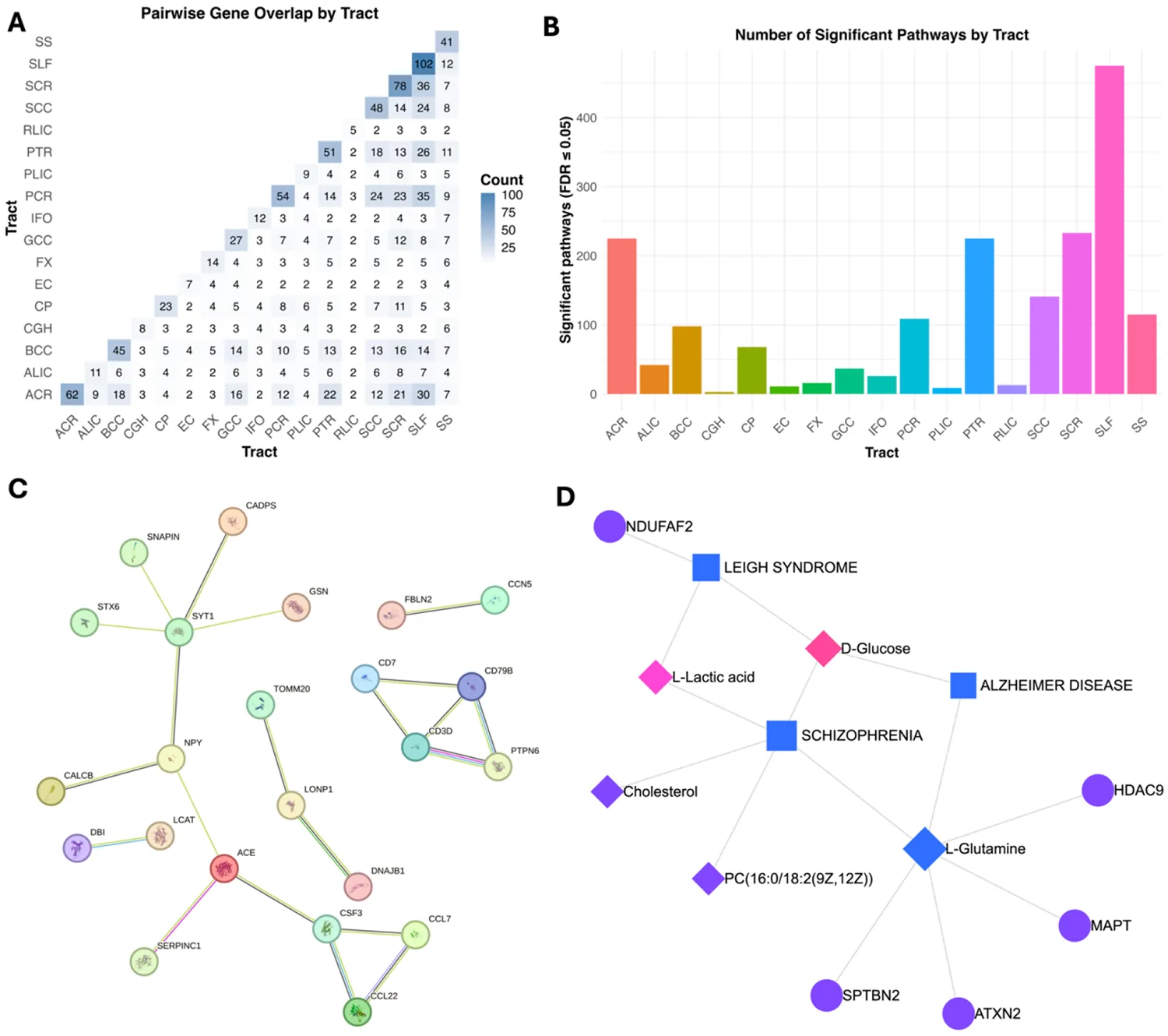
Heatmap displaying overlapping gene sets identified for each of the 17 observed white matter tracts. Genes were identified via FUMA using significant independent genetic loci associated with FA parameters. (B) Number of enriched biological pathways by tract, based on genes significantly associated with FA. Pathway enrichment was conducted using g:Profiler. (C) Protein-protein interaction network for identified proteomics features significantly associated with FA parameters, constructed using the STRING platform. (D) Selected multi-omics sub-network of metabolite-gene-disease features, constructed using MetaboAnalyst. Included metabolomics and gene features were significantly associated with FA parameters in our GWAS and pheWAS.

We then mapped the 315 independent genome-wide significant variants from the discovery GWAS together with 97 proteomic features from the PheWAS to 237 genes using FUMA [20]. Gene-set enrichment with g:Profiler [21] revealed significant over-representation of pathways related to neurogenesis, cytoplasmic processes, negative chemotaxis, and actin binding (adjusted P < 0.001). Many of these pathways play central roles in neural connectivity, axonal remodeling, and cytoskeletal dynamics—biological processes fundamental to white-matter integrity and consistent with earlier large-scale imaging-genetics studies [13, 22]. When enrichment was repeated for tract-level FA-associated genes, pronounced regional heterogeneity emerged (Fig. 5b), with the superior longitudinal fasciculus (SLF) and anterior corona radiata (ACR) showing the greatest number of enriched pathways. These patterns suggest that specific association and projection pathways may act as molecular hubs, where diverse genetic programs converge to shape microstructural organization.

To examine functional relationships among implicated genes, we constructed protein–protein interaction networks using STRING [23]. The resulting networks showed strong over-representation of extracellular and signaling-related functions (Fig. 5c). FA-associated proteins were enriched for protein-binding and signaling-receptor-binding functions (FDR < 0.05) and were preferentially localized to extracellular regions and vesicular compartments, including dense-core granules involved in neuropeptide release. These findings highlight an integrated molecular landscape dominated by secreted and receptor-binding proteins, suggesting that intercellular communication, neurovascular interactions, and neuroimmune signaling contribute to the maintenance of white-matter microstructure. Enrichment of secreted and signal-related UniProt keywords, together with blood-expressed proteins, further implies that circulating signaling molecules may influence white matter through peripheral-to-central pathways.

To integrate genetic, proteomic, and metabolic layers, we constructed a gene–metabolite–disease interaction network using MetaboAnalyst [24] (Fig. 5d). FA-associated genetic and proteomic features were mapped to known metabolic pathways and linked to significantly associated metabolites through curated gene–metabolite relationships. The resulting network revealed several highly interconnected modules spanning metabolic and signaling pathways involved in neuronal maintenance, oxidative stress, and lipid metabolism—core processes underlying white-matter integrity. Notably, Alzheimer’s disease (AD) and schizophrenia (SCZ) emerged as central nodes connected to multiple genes and metabolites across omics layers, indicating shared molecular substrates between the genetic regulation of FA and metabolic perturbations implicated in neurodegeneration and psychiatric disorders.

Collectively, these multi-omics and network analyses bridge genome-wide variation, molecular signaling, and macroscopic white-matter structure, revealing a systems-level architecture in which coordinated lipid metabolism, cytoskeletal organization, and extracellular signaling converge to shape human white-matter microstructure. This integrated framework highlights biological pathways through which genetic risk may propagate from molecular systems to large-scale neural circuits relevant to disease.

## Discussion

This study provides a comprehensive and spatially resolved view of the genetic and molecular architecture underlying human white-matter microstructure. By integrating GWAS of location-level diffusion metrics (FA, MD, AD) with causal inference and multi-omics profiling, we establish a multiscale framework that connects common genetic variation to tissue-level organization and systemic molecular processes. Our results show that genetic influences on white-matter integrity are both widespread and spatially structured, reveal tract-specific pathways of causal influence on cognitive and neuropsychiatric traits, and uncover convergent molecular signatures linking lipid metabolism, cytoskeletal remodeling, and extracellular signaling to the maintenance of white-matter architecture.

The spatially organized and highly replicable heritable patterns suggest that white-matter structure is governed by stable, genetically encoded principles of neural wiring. Regions with the strongest heritable signals, particularly major projection and callosal tracts, correspond to long-range pathways that provide essential communication infrastructure across the brain. The identification of numerous independent loci distributed along these systems supports a view of white-matter development and maintenance as a polygenic, systems-level process, shaped by pathways involved in axonal growth, myelination, neurovascular coupling, and glial homeostasis. The extensive polygenic overlap between FA and traits such as AD, depression, and cognitive performance further highlights white-matter microstructure as a central biological axis linking genetic risk for neurodegenerative and psychiatric conditions to the integrity of large-scale communication networks. The tract-specific causal effects observed for AD and cognitive traits reinforce this view, suggesting that maintaining the integrity of key projection pathways may contribute to resilience in cognitive aging.

The convergence of genetic, proteomic, and metabolomic associations onto overlapping anatomical substrates provides molecular context for these macrostructural patterns. Enrichment of lipid-metabolic, cytoskeletal, and extracellular signaling pathways points to coordinated processes of membrane maintenance, axonal transport, and intercellular communication as core molecular determinants of white-matter organization. The observation that circulating metabolites—particularly those related to lipid transport and oxidative stress—show spatial concordance with genetic association hotspots indicates that systemic physiology exerts measurable influence on neural microstructure. This aligns with emerging models in which neurovascular and neuroimmune pathways couple peripheral metabolic state to central tissue maintenance. More broadly, the multi-omics networks identified here demonstrate that genomic, proteomic, and metabolic variation converge on a shared architectural scaffold, linking molecular homeostasis and systemic physiology to large-scale connectivity and brain health.

Despite these strengths, several limitations remain. The sample consists primarily of middle-aged adults of white British ancestry, and extending analyses across diverse ancestries, developmental periods, and aging trajectories will be essential. Although FA provides a sensitive index of microstructural variation, it is not fully specific; integrating advanced diffusion models and myelin-sensitive MRI would refine biological interpretation. Multi-omics integration was constrained by modality-specific sample sizes and measurement noise, motivating larger harmonized datasets and causal-mediation frameworks to clarify the directional relationships among genetic, molecular, and imaging layers. Finally, while tract-level Mendelian randomization highlights candidate causal pathways, experimental validation and longitudinal imaging will be necessary to confirm these mechanisms and determine how genetic and systemic variation jointly shape white-matter health across the lifespan.

## Supplementary Material

Supplement 1

## Figures and Tables

**Figure 1.**
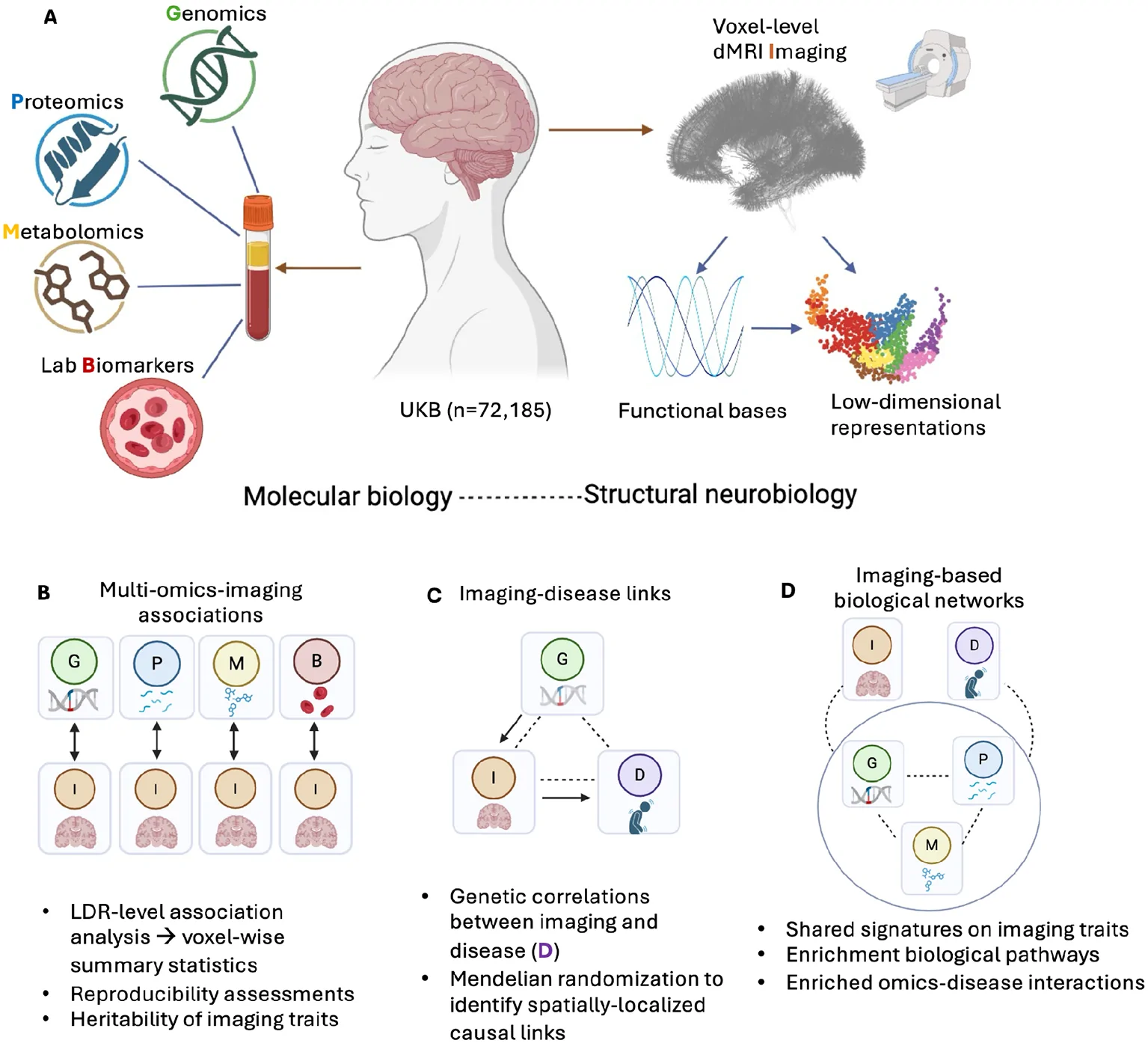
Schematic overview of study and dataset.
