# Supplementary material for: A spatially resolved genomic–molecular atlas of human white-matter microstructure
Source: medRxiv. 2026 Jul 9:2026.07.06.26357381. Preprint. [Version 1] doi: 10.64898/2026.07.06.26357381 (PMC13370573; doi:10.64898/2026.07.06.26357381)
Supplement: Supplementary file 1 [file media-1.pdf]

Supplementary Materials for

A spatially resolved genomic-molecular atlas of human white-matter microstructure

Contents

1 Materials and Methods **3**

1.1 Study cohorts and imaging data . . . 3
1.2 Image acquisition and preprocessing . . . 3
1.3 Functional PCA and low-dimensional representations . . . 4
1.4 Genotype data and quality control . . . 5
1.5 GWAS of low-dimensional representations . . . 5
1.6 Voxel-level reconstruction of GWAS effects . . . 6
1.7 Spatial post-screening and locus definition . . . 6
1.8 Replication framework . . . 7
1.9 Heritability estimation . . . 7
1.10 Genetic correlation analysis . . . 8
1.11 Mendelian randomization analysis . . . 8
1.12 Colocalization analysis . . . 9
1.13 Multi-omics association analysis . . . 9
1.14 Cross-omics spatial concordance . . . 10
1.15 Biological enrichment and network integration . . . 11
1.16 Cross-modality comparison framework . . . 12
1.17 Sensitivity analyses . . . 12

2 Supplementary Results **13**

2.1 Properties of low-dimensional representations . . . 13
2.2 Quality control and calibration . . . 13
2.3 Voxel-wise heritability patterns . . . 14
2.4 Spatial GWAS atlases . . . 15
2.5 Replication of white-matter loci . . . 16
2.6 Genetic correlation results . . . 17
2.7 Mendelian randomization results . . . 18
2.8 Multi-omics associations and cross-omics spatial concordance . . . 19
2.9 Biological enrichment and networks . . . 20

3 References **21**

4 Supplementary Figures **24**
Figure S1: CBSS white-matter atlas used for fiber-level analyses 24
Figure S2: Properties of low-dimensional representations (LDRs) 24
Figure S3: Quality control and calibration of voxel-level GWAS 24
Figure S3: Quality control and calibration of voxel-level GWAS 24
Figure S5: Spatial atlases of voxel-level genetic effects 24
Figure S6: Colocalization at 5q13.3 (Type 2 diabetes) 24
Figure S7: Colocalization at 11p11.2 (Alzheimer's disease) 24
Figure S8: Colocalization at 17p11.2 (Stroke) 24
Figure S9: Colocalization at 6p25.1 (Glomerular filtration rate) 24
Figure S10: Colocalization at 19q13.32 (Coronary atherosclerosis) 24
Figure S11: Cross-omics phenotypic and spatial correlation maps 24
Figure S12: Fiber-level genetic correlation atlases with brain-related traits . 24
Figure S13: Spatial replication of white-matter loci across tracts 24
Figure S14: Genomic distribution of white-matter loci across diffusion MRI modalities 24
Figure S15: Genomic distribution of FA loci across UKB and ABCD cohorts 24

1 Materials and Methods

1.1 Study cohorts and imaging data

This study utilized diffusion magnetic resonance imaging (dMRI) and genetic data from the UK Biobank (UKB), a large population-based cohort study comprising over 500,000 participants aged 40–69 years at recruitment (Sudlow et al. 2015). Imaging data were obtained from the UKB imaging substudy, which includes multimodal brain MRI acquired using standardized protocols across multiple assessment centers (Miller et al. 2016). For the primary analyses, we included 56,313 individuals of European ancestry from UKB Phases 1–6 with both imaging and genetic data available after quality control. To minimize population stratification and ensure homogeneity in genetic analyses, we restricted analyses to unrelated individuals of British ancestry as defined by UKB genetic principal components and self-reported ethnicity Bycroft et al. (2018). Participants with missing imaging data, poor image quality, or failing genetic quality control were excluded.

The discovery and replication analyses were conducted using non-overlapping subsets of the UK Biobank imaging cohort. The discovery cohort consisted of 56,313 individuals from UK Biobank Phases 1–6, while the replication cohort included 15,872 individuals from UK Biobank Phase 7. This design enabled independent validation of genetic associations identified in the discovery stage.

Diffusion MRI data were acquired using a standardized protocol on 3T Siemens Skyra scanners with a 32-channel head coil (Miller et al. 2016). The diffusion protocol included multiple diffusion-weighted directions and b-values, enabling estimation of diffusion tensor imaging (DTI) measures such as fractional anisotropy (FA), mean diffusivity (MD), and axial diffusivity (AD). Imaging data were processed using the connectivity-based spatial statistics (CBSS) pipeline (Li et al. 2026), which projects diffusion measures onto a white-matter skeleton and constructs a high-resolution representation of white-matter fiber architecture across subjects. To assess robustness and generalizability of findings, additional external imaging datasets—including the Human Connectome Project-Aging (HCP-A) and the Adolescent Brain Cognitive Development (ABCD) study—were processed using analogous pipelines for selected validation analyses (Bookheimer et al. 2019; Casey et al. 2018).

1.2 Image acquisition and preprocessing

Following projection of diffusion tensor imaging (DTI) measures onto the white-matter skeleton, all subjects were nonlinearly registered to MNI152 space (Mazziotta et al. 2001). Harmonization procedures were applied to mitigate site-specific variation across imaging centers (Li et al. 2026).

The white-matter skeleton was partitioned into 6,090 spatially contiguous fiber segments, each represented by 100 ordered voxels, yielding a total of 609,000 voxel-level measurements per subject. We included all 6,090 fiber clusters from the CBSS atlas in the genetic analyses, which is larger than the 5,723 clusters used in Li et al. (2026), where pathways with low mean fiber count or test–retest reliability were excluded. For GWAS, low phenotypic reproducibility primarily attenuates genetic effect sizes

rather than generating false-positive associations, and the large UK Biobank sample compensates for this reduced signal. By contrast, the thresholds applied in Li et al. (2026) reflect the stricter stability requirements of normative growth-chart modeling, where sparse or poorly reproducible pathways yield unstable age-trajectory estimates.

To reduce computational burden and improve statistical efficiency, we performed hierarchical clustering to group fibers into 430 fiber clusters. Clusters were constructed such that all constituent fibers belonged to the same functional network pair and were within 30 mm of one another, preserving both anatomical proximity and functional coherence. Hierarchical clustering was performed using Ward's minimum variance method applied to the spatial coordinates of fiber centroids in MNI152 space. The clustering proceeded in two stages: fibers were first grouped by functional network pair (as defined within the CBSS framework), and within each network pair, agglomerative clustering was applied subject to the constraint that no two fibers in the same cluster could have centroids more than 30 mm apart. The final partition into 430 clusters was determined by a minimum cluster-size criterion of 5 fibers, below which clusters were merged with their nearest neighbor. Clusters ranged in size from 5 to 47 fibers (median: 12), with larger clusters concentrated in major projection and callosal pathways.

For downstream interpretation, fiber clusters were mapped to canonical white-matter regions of interest defined by the JHU ICBM-DTI-81 atlas (Oishi et al. 2008). Specifically, for each cluster, we computed the mean distance between its component fibers and each of 26 atlas-defined tract regions, assigning clusters to the nearest tract when the distance was less than 10 mm. Using this procedure, 399 of the 430 clusters were assigned to one of 17 major white-matter tracts, while the remaining clusters were left unassigned. In addition, all clusters were annotated according to 66 functional network pairs (corresponding to 13 unique functional networks) as defined within the CBSS framework (Jiang et al. 2026). Distances were computed as the mean Euclidean distance between the voxel coordinates of all fibers within a cluster and the binary mask of each JHU atlas ROI, using a nearest-voxel approach. The 10 mm threshold was selected to balance anatomical specificity against coverage: thresholds below 8 mm left more than 20% of clusters unassigned, while thresholds above 12 mm produced ambiguous assignments in regions of closely adjacent tracts such as the corona radiata and internal capsule. The 26 JHU atlas regions were consolidated into 17 canonical tracts by merging bilateral homologs (e.g., left and right corticospinal tract) and subregions of the same structure (e.g., genu, body, and splenium of the corpus callosum were retained as distinct tracts). The 31 unassigned clusters, primarily located at tract intersections or in deep white matter regions poorly covered by the JHU atlas, were retained in all GWAS and heritability analyses but excluded from tract-level aggregation and visualization.

1.3 Functional PCA and low-dimensional representations

Using the preprocessed imaging data, we applied functional principal component analysis (fPCA) to obtain low-dimensional representations (LDRs) of voxel-level fractional anisotropy (FA) within each fiber cluster (Shang 2014; Jiang et al. 2026). Functional basis functions were estimated using UKB Phase 1–6 data via a local linear smoothing

approach (Fan 2018), after which cluster-specific FA signals from all subjects (Phases 1–6) were projected onto these bases to obtain LDR scores.

Because fiber clusters vary in size and spatial covariance structure, the number of retained components was selected adaptively for each cluster to capture 80–90% of total variance while maintaining high reconstruction fidelity (correlation 0.85–0.95 between original and reconstructed signals). The resulting eigenvalues summarize the variance explained by each component and characterize the intrinsic dimensionality of voxel-level variation within each cluster.

To account for spatial correlation in downstream inference, we estimated the effective number of independent voxels using the eigenvalue spectrum,

$$M_{\text{eff}} = \left(\sum_{k=1}^{K} \lambda_k\right)^2 \Bigg/ \sum_{k=1}^{K} \lambda_k^2,$$

which quantifies the degree of redundancy among voxel measurements. Summing across clusters yielded approximately 5,479 effective independent voxels across the white-matter skeleton. This quantity was used to calibrate multiple testing correction and inform significance thresholds in subsequent voxel-level analyses. A summary of cluster-level fPCA characteristics is provided in Table SX.

1.4 Genotype data and quality control

Genotyped and imputed genetic variant data were obtained from the UK Biobank. Quality control procedures were applied to individuals with both imaging and genetic data available following standard GWAS protocols (Turner et al. 2011; Marees et al. 2018). We excluded individuals with more than 10% missing genotypes. Variants were filtered to remove those with minor allele frequency less than 0.01, missing genotype rate greater than 10%, or deviation from Hardy–Weinberg equilibrium at $P < 1 \times 10^{-7}$ (Turner et al. 2011; Marees et al. 2018). For imputed variants, we additionally required an imputation INFO score greater than 0.8, consistent with the standard practice in large-scale GWAS (Marees et al. 2018). All quality control procedures were implemented using established GWAS tools, including PLINK (Purcell et al. 2007; Chang et al. 2015).

After quality control, approximately 7.8 million variants remained. Following the removal of strand-ambiguous variants, 6.8 million variants were retained for downstream genome-wide association analyses. In addition, approximately 460,000 directly genotyped SNPs were available from the unimputed dataset. For validation analyses, independent imaging cohorts from the Human Connectome Project-Aging (HCP-A) and the Adolescent Brain Cognitive Development (ABCD) study were processed using comparable pipelines.

1.5 GWAS of low-dimensional representations

We conducted genome-wide association studies (GWAS) on 8,868 low-dimensional representations (LDRs) of diffusion tensor imaging (DTI) phenotypes using the

Representation learning-based Voxel-level Genetic Analysis (RVGA) framework implemented in the Highly Efficient Imaging Genetics (HEIG) tool (Jiang et al. 2026). The discovery analysis included 56,313 individuals of British ancestry from UKB Phases 1–5, and replication analyses were performed in an independent cohort of 14,965 individuals from UKB Phase 7.

For each LDR, we fit linear regression models testing the association between genetic variants and LDR values (Purcell et al. 2007), adjusting for age, sex, age×sex, age^2, $\text{age}^2\times$sex, 40 genetic principal components, and assessment center. To account for sample relatedness and polygenic background effects, we applied a two-step ridge regression procedure (Hoerl and Kennard 1970). First, genotyped variants were partitioned into approximately 100 blocks of ∼5,000 variants, and ridge regression models were fit within each block to predict LDR values. Second, predictions from all blocks were combined and used in a leave-one-chromosome-out (LOCO) framework (Loh et al. 2015) to generate chromosome-specific polygenic scores, which were included as offsets in GWAS to control for relatedness.

The shrinkage parameter in the ridge regression was selected via 5-fold cross-validation, reflecting the contribution of polygenic effects to each LDR. This approach improves statistical calibration and power while maintaining computational scalability across high-dimensional imaging phenotypes (Jiang et al. 2026).

1.6 Voxel-level reconstruction of GWAS effects

Following LDR-level GWAS, voxel-level association statistics were reconstructed by integrating LDR summary statistics with the corresponding fPCA basis functions and LDR variance–covariance matrices, as described in the RVGA framework (Jiang et al. 2026). This procedure maps genetic effects estimated in the reduced LDR space back to the original voxel space, enabling spatially resolved inference across the white-matter skeleton.

Specifically, for each variant, voxel-level effect sizes and test statistics were obtained as linear combinations of LDR-level associations weighted by the fPCA loadings. This reconstruction preserves the covariance structure induced by dimensionality reduction and allows efficient recovery of high-resolution genetic association patterns without performing voxel-wise GWAS directly.

1.7 Spatial post-screening and locus definition

To prioritize robust and spatially coherent associations, we applied a post-screening procedure to voxel-level GWAS results. For each fiber cluster, null distributions of voxel cluster size (defined as the number of voxels exceeding $P < 1 \times 10^{-5}$) were generated using wild bootstrap sampling ($B = 50$).

We first identified unique significant SNPs in the discovery cohort at a genome-wide threshold of 5×10^{-8} (Chen et al. 2021) adjusted by the total effective number of independent voxels ($M_{\text{eff}} = 5479.48$). For these SNPs, voxel-level associations were reconstructed at $P < 1 \times 10^{-5}$ and compared against cluster-specific null distributions. SNPs were retained if their associated voxel clusters exceeded the threshold defined by the quantile $1 - (1 - 1 \times 10^{-5})^K$, where K is the effective number of voxels

within the cluster. This procedure yielded variants that were both genetically significant and spatially enriched. Independent loci were then defined using a three-stage linkage disequilibrium (LD) clumping procedure (Purcell et al. 2007): (i) cluster-level independent significant SNPs were identified using $r^2 > 0.6$ within a 1 Mb window; (ii) cluster-level lead SNPs were obtained by further clumping at $r^2 > 0.1$ within 500 kb; and (iii) tract-level lead SNPs were defined by merging signals across clusters using the same LD threshold.

1.8 Replication framework

Replication analyses were conducted at the tract level using independent UKB Phase 7 data. For each tract-level lead SNP identified in discovery, we searched for SNPs within a ± 250 kb window in the replication cohort. If a corresponding SNP passed post-screening criteria in replication, the locus was considered genetically replicated.

Spatial replication was evaluated by comparing voxel-level association patterns between discovery and replication. For each locus, we defined the set of associated voxels as the union of voxels across all significant SNPs within the ± 250 kb region. The proportion of discovery-associated voxels that were also significant in replication was then computed to quantify spatial concordance.

In the UKB Phase 7 replication cohort, voxel-level associations were reconstructed and evaluated at a threshold of $P < 1 \times 10^{-4}$, reflecting common practice in imaging GWAS studies that adopt moderately relaxed thresholds in follow-up analyses of high-dimensional neuroimaging phenotypes (Elliott et al. 2018; Stein et al. 2010). For external validation in the ABCD dataset, we used a more permissive threshold of $P < 1 \times 10^{-3}$ to account for reduced sample size and differences in cohort characteristics. In both settings, cluster-size thresholds were determined using bootstrap-based null distributions (Jiang et al. 2026). Replication performance was summarized by the number of loci exhibiting any spatial overlap ($> 0\%$) and those with substantial overlap ($> 50\%$) between discovery and replication.

1.9 Heritability estimation

We estimated voxel-level SNP heritability using the RVGA estimator, which enables efficient inference for high-dimensional imaging phenotypes. Briefly, genetic covariance was first estimated in the LDR space and then projected to the voxel level using the corresponding functional basis functions to obtain voxel-wise heritability estimates. Analytic standard errors were derived following the procedure described in Jiang et al. (2026). For quality control, we excluded voxel-level estimates with out-of-bounds heritability values (e.g., negative estimates or values exceeding 1) or inflated standard errors. Statistical significance was determined using a Bonferroni correction based on the total effective number of independent voxels ($M_{\text{eff}} = 5479.48$; $P < 0.05/M_{\text{eff}}$).

To facilitate anatomical interpretation, voxel-wise heritability estimates were aggregated to the ROI level for 17 major white-matter tracts and 66 functional network pairs. For each ROI, we summarized the mean heritability and the proportion of

significant voxels, and computed ROI-level p-values using the aggregated Cauchy association test (ACAT; Liu et al. (2019)), which provides valid inference under arbitrary correlation among input statistics.

1.10 Genetic correlation analysis

We estimated voxel-level genetic correlations between white-matter microstructure and 14 external phenotypes using publicly available GWAS summary statistics for each trait. These included attention-deficit/hyperactivity disorder (ADHD; Demontis et al. 2023), bipolar disorder (Mullins et al. 2021), major depressive disorder (Wray et al. 2018), insomnia (Watanabe et al. 2022), autism spectrum disorder (ASD; Grove et al. 2019), schizophrenia (Trubetskoy et al. 2022), and Alzheimer's disease (Wightman et al. 2021). We additionally included cognitive performance and educational attainment (Lee et al. 2018), as well as intelligence (Savage et al. 2018). Personality-related traits were derived from a large-scale GWAS of neuroticism, including overall neuroticism, neuroticism subclusters (depressed affect and worry), and depression (Nagel et al. 2018).

Genetic correlation analyses were performed using these summary statistics in a two-sample framework implemented in HEIG (Jiang et al. 2026). Specifically, GWAS summary statistics for LDRs were combined with publicly available summary statistics for external traits to estimate genetic correlations in the LDR space. Alleles were aligned to reference LD panels, and linkage disequilibrium (LD) matrices and their inverses were constructed using regularization levels of 90% and 85%, respectively, to ensure numerical stability. Genetic correlation estimates were then projected to voxel space using the fPCA basis functions, yielding spatial maps of shared genetic architecture. For interpretation, voxel-level genetic correlations were aggregated to tract-level summaries.

1.11 Mendelian randomization analysis

We investigated potential causal relationships between white-matter microstructure and the same set of 14 external phenotypes using two-sample Mendelian randomization (MR). Genetic instruments were selected from LDR-level GWAS summary statistics by retaining variants reaching genome-wide significance ($P < 5 \times 10^{-8}$) and applying LD-based clumping ($r^2 < 0.01$, 10 Mb window) using the 1000 Genomes European reference panel to ensure independence.

Exposure and outcome datasets were harmonized using the TwoSampleMR package (Hemani et al. 2018), which standardizes allele alignment across traits. We applied multiple MR estimators, including inverse variance weighted (IVW), MR-Egger, weighted median, weighted mode, simple mode, and MR-GRIP, to assess robustness to pleiotropy and model assumptions.

MR analyses were performed at the LDR level and subsequently aggregated to the tract level to improve interpretability and statistical power. For each tract–trait–method combination, p-values across LDRs were combined using the aggregated Cauchy association test (ACAT) (Liu et al. 2019). Multiple testing was controlled using the Benjamini–Hochberg procedure across all tract $\times$ trait $\times$ method

combinations at a false discovery rate of 5%. To ensure directional consistency, we required that contributing LDR effects within each tract exhibited concordant signs; associations with inconsistent directions were excluded. For visualization and interpretation, we focused on tract–trait associations that (i) passed FDR ≤ 0.05 under the fixed-effect IVW model and (ii) were supported by at least two additional MR methods (for a total of ≥ 3 methods) with concordant directionality and FDR ≤ 0.05.

1.12 Colocalization analysis

To assess whether white-matter microstructure and external traits share common causal variants, we performed Bayesian colocalization analyses at each discovery locus using the *coloc* R package (Giambartolomei et al. 2014). For each locus, we selected a representative voxel defined as the voxel with the strongest association (minimum P-value) with the lead SNP. Voxel-level association statistics were then reconstructed genome-wide for this representative voxel and merged with GWAS summary statistics for a panel of external traits, including neuropsychiatric, cognitive, and cardiometabolic phenotypes (Table SX). SNP-wise effect estimates, standard errors, minor allele frequencies, and sample sizes were aligned across datasets prior to analysis.

Colocalization was performed using the approximate Bayes factor method (*coloc.abf*), which evaluates five competing hypotheses: no association (H0), association with only the imaging trait (H1), association with only the external trait (H2), two distinct causal variants (H3), or a shared causal variant (H4). The imaging phenotype (voxel-level FA) was modeled as a quantitative trait, while external traits were specified as quantitative or case-control according to their study design, with case prevalence incorporated for binary traits. For each locus–trait pair, we computed posterior probabilities for all hypotheses and used the posterior probability of the shared causal variant model (PP.H4) to quantify evidence for colocalization.

To prioritize robust signals, we restricted analyses to locus–trait pairs exhibiting spatial replication between discovery and replication cohorts (50% overlap of associated voxels). Among these, we defined strong colocalization as PP.H4 0.8 with PP.H4 ¿ PP.H3, ensuring support for a shared rather than distinct causal signal. Results were summarized across loci and traits to generate prioritized lists of locus–trait pairs ranked by PP.H4 and spatial overlap. These results were used to produce summary visualizations, including distributions of PP.H4, heatmaps of locus–trait colocalization, trait-level counts of colocalized loci, and locus-specific regional association plots illustrating concordance between imaging and external traits.

1.13 Multi-omics association analysis

Voxel-level multi-omics association analyses were conducted to identify molecular correlates of white-matter microstructure using a framework parallel to the GWAS described above. For each omics modality—proteomics, metabolomics, and clinical laboratory biomarkers—we obtained data from UK Biobank (UKB) subsets, including plasma proteomics from the UKB Pharma Proteomics Project (UKB-PPP; $n = 5{,}614$; Sun et al. (2023)), nuclear magnetic resonance (NMR)–based metabolomics from

Nightingale Health (n = 26,412), and clinical laboratory measurements from UKB assessment center blood panels (n = 18,689).

We leveraged the same functional principal component analysis (fPCA) bases and low-dimensional representations (LDRs) of fractional anisotropy (FA) derived from unrelated UKB Phase 1–6 participants (see Functional PCA). For each modality, linear association models were fit at the LDR level within the subset of subjects with available omics data, regressing each LDR on individual molecular features while adjusting for the same covariates used in the GWAS.

Voxel-level association statistics were reconstructed from LDR-level results using the shared fPCA basis functions, enabling direct comparison with GWAS-derived association maps. Multiplicity correction accounted for correlation across both voxels and molecular features. Specifically, the effective number of omics features was estimated from the eigenvalue spectrum of the feature–feature correlation matrix, and the effective number of voxels (5,479.48) was carried forward from the GWAS calibration. The overall correction factor was defined as the product of these two effective numbers.

To further control false positives arising from spatial correlation, we applied a spatial-extent correction analogous to the GWAS procedure. We generated 50 wild-bootstrap replicates ($B = 50$) by resampling LDR residuals within subjects and reconstructing voxel-level association maps under the null. Empirical null distributions of cluster sizes (defined as the number of voxels exceeding $P < 1 \times 10^{-5}$) were used to derive cluster-wise significance thresholds. Only voxel clusters exceeding these thresholds were retained as statistically and spatially significant.

This framework yielded modality-specific multi-omics association atlases, mapping proteomic, metabolomic, and clinical biomarker effects onto white-matter microstructure at voxel-level resolution.

1.14 Cross-omics spatial concordance

To characterize shared spatial signatures across molecular features and omics modalities, we quantified similarity in voxel-wise association maps derived from the multi-omics analyses. For each feature, we used voxel-level Z-score maps reconstructed from LDR-level association models (see Multi-omics association analysis). Pairwise spatial similarity was assessed by computing Pearson correlation coefficients (r) between all pairs of Z-score maps across the 609,000 voxels within the white-matter skeleton. Analyses were performed both within each modality and across modalities to evaluate within- and cross-layer concordance in spatial association patterns.

To summarize overall correspondence, we computed absolute correlation coefficients ($|r|$) and reported their mean values within and between modalities. These summaries capture the strength of spatial similarity independent of effect direction. To complement correlation-based measures, we also quantified spatial overlap of statistically significant regions. Voxel-wise maps were thresholded at a false discovery rate (FDR) of 0.05 using the joint effective-number correction described above, and binary significance masks were constructed for each feature. For each pair of features,

we computed the Dice coefficient, defined as

$$\text{Dice} = \frac{2|A \cap B|}{|A| + |B|},$$

where A and B denote the sets of significant voxels for the two features. The Dice coefficient provides a measure of spatial co-localization that accounts for differences in signal extent. Together, correlation- and Dice-based metrics characterize the degree of convergence or divergence in molecular association patterns across omics layers.

1.15 Biological enrichment and network integration

To contextualize genetic and molecular associations identified in the voxel-level GWAS and multi-omics analyses, we performed integrative enrichment and network analyses spanning gene, protein, metabolite, and disease layers. For GWAS-derived signals, voxel-level association statistics were summarized at each locus by selecting a representative voxel defined as the voxel with the strongest association (minimum P-value) with the tract-level lead SNP. Genome-wide summary statistics for these representative voxels were used as input for functional annotation using FUMA (v1.5.7; Watanabe et al. (2017)).

Genome-wide significant SNPs were mapped to genes based on cis-eQTL relationships from GTEx v8 (GRCh37/hg19), retaining SNP–gene associations meeting an FDR threshold of 0.05. Each independent variant was thus linked to one or more candidate genes through tissue-specific expression associations, primarily in brain tissues. Proteomic features from the UKB Pharma Proteomics Project were mapped to their corresponding encoding genes prior to downstream analyses, enabling a unified gene-level representation across GWAS and proteomic results.

Gene-set enrichment analyses were performed using g:Profiler (v0.2.2; Kolberg et al. (2023)) across multiple annotation databases, including Gene Ontology (Biological Process, Molecular Function, Cellular Component), KEGG, and Reactome. Enrichment analyses were conducted separately for gene sets derived from GWAS and proteomic associations and subsequently integrated for interpretation. Multiple testing was controlled using the g:SCS method, and pathways with adjusted $P < 0.05$ were considered significant.

To assess functional connectivity among identified genes and proteins, protein–protein interaction (PPI) networks were constructed using STRING (v12.0; Szklarczyk et al. (2019)). Only experimentally supported interactions with confidence score > 0.4 were retained. Network enrichment statistics provided by STRING were used to evaluate whether the observed connectivity exceeded random expectation. Networks were annotated using Gene Ontology terms and UniProt keywords and visualized to highlight clusters enriched for extracellular signaling, synaptic processes, and metabolic pathways.

To integrate molecular layers, we constructed joint gene–metabolite–disease networks using the Network Analysis module in MetaboAnalyst (v5.0; Pang et al. (2021)). Gene–metabolite relationships were obtained from the Human Metabolome Database (HMDB), and disease annotations were assigned through MetaboAnalyst's internal

ontology framework. All identified entities were included without pathway pre-filtering. The resulting networks were analyzed to identify connected components and highly interconnected subnetworks representing shared biological mechanisms across omics layers, including processes related to neuronal maintenance, oxidative stress, and lipid metabolism.

1.16 Cross-modality comparison framework

To investigate shared and distinct genetic architecture across diffusion modalities, we extended selected analyses from fractional anisotropy (FA) to mean diffusivity (MD) and axial diffusivity (AD). All preprocessing, dimensionality reduction, GWAS, and voxel-level reconstruction procedures described above were applied analogously to each modality to ensure comparability of results.

We considered three complementary questions. First, to assess whether discovery loci preferentially localize to similar tract systems across modalities, we mapped tract-level lead SNPs identified for each modality to their associated white-matter regions and compared patterns of tract-level enrichment. Overlap across modalities was evaluated by identifying loci mapped to the same tract systems and summarizing the distribution of shared and modality-specific tract associations.

Second, to evaluate whether spatial association patterns are conserved across modalities, we compared voxel-level association maps for corresponding loci across FA, MD, and AD. For each locus, voxel-wise Z-score maps were reconstructed for each modality, and spatial concordance was quantified using Pearson correlation coefficients and overlap metrics (e.g., Dice coefficients) within the locus-specific regions.

Third, to determine whether shared locus signals arise from the same underlying causal variant, we performed cross-modality colocalization analyses using the same Bayesian framework described above. For loci detected in multiple modalities, summary statistics were compared within each locus to estimate posterior probabilities of shared versus distinct causal variants. Together, these analyses characterize the extent to which genetic influences on white-matter microstructure are conserved or modality-specific across diffusion measures.

1.17 Sensitivity analyses

We performed a series of sensitivity analyses to evaluate the robustness of key findings to analytic choices made in the reconstruction, replication, and downstream prioritization steps. In particular, because voxel-level replication depends on the threshold used to reconstruct association maps in the validation cohort, we repeated replication analyses using alternative significance thresholds of $P < 1 \times 10^{-3}$ and $P < 1 \times 10^{-2}$ in addition to the primary threshold of $P < 1 \times 10^{-4}$. We then re-computed genetic replication counts, spatial overlap summaries, and the proportion of loci meeting the predefined replication criteria.

To assess robustness of the spatial replication framework, we also examined alternative definitions of spatial overlap between discovery and replication signals, including any nonzero overlap and more stringent overlap thresholds beyond the primary criterion. For colocalization analyses, we evaluated whether the set of prioritized locus–trait

pairs was sensitive to the posterior probability cutoff used to define strong evidence of a shared causal variant. Across these analyses, our primary conclusions were based on patterns that remained qualitatively consistent under reasonable variations in thresholding and prioritization criteria.

2 Supplementary Results

2.1 Properties of low-dimensional representations

We evaluated the statistical and structural properties of the low-dimensional representations (LDRs) derived from functional principal component analysis (fPCA) to ensure adequate capture of voxel-level variation in white-matter microstructure. Across the 430 fiber clusters, the number of retained components was selected adaptively to explain 80–90% of total variance, resulting in a total of 8,868 LDRs.

Reconstruction accuracy was assessed by comparing the original voxel-level signals with their fPCA-based reconstructions. Across clusters, the correlation between original and reconstructed signals ranged from 0.85 to 0.99, with a mean of 0.94, indicating that the LDRs preserved the majority of voxel-level information. The number of retained components varied across clusters, reflecting heterogeneity in spatial complexity and covariance structure of white-matter regions.

The eigenvalue spectra of the fPCA decompositions exhibited rapid decay across clusters, indicating that a relatively small number of components captured most of the variability in voxel-level signals. These eigenvalues were further used to estimate the effective number of independent voxels within each cluster. Summing across clusters yielded approximately 5,479 effective independent voxels across the white-matter skeleton, substantially reducing the dimensionality relative to the original 609,000 voxel measurements.

2.2 Quality control and calibration

We evaluated the statistical calibration of the GWAS analyses at both the LDR and voxel levels to ensure robustness of downstream inference. For each LDR, quantile–quantile (QQ) plots were examined to compare observed and expected distributions of test statistics under the null hypothesis. Across LDRs, the genomic inflation factor (λ_{GC}) was close to 1 (mean = 1.046, range = 0.983–1.185), indicating well-calibrated association statistics with no evidence of systematic inflation beyond that expected from polygenic signal.

To account for sample relatedness and population structure, we implemented a two-step ridge regression procedure with leave-one-chromosome-out (LOCO) adjustment, which effectively removed polygenic background effects prior to association testing. This approach improved calibration and ensured appropriate control of type I error across high-dimensional imaging phenotypes.

We further assessed calibration of voxel-level association statistics reconstructed from LDR-level results. The distribution of voxel-level test statistics was consistent with expectations under the null, with no evidence of artifactual inflation introduced

by the reconstruction procedure. Together, these results demonstrate that both LDR-level and voxel-level analyses were well calibrated and suitable for downstream genetic and spatial inference.

2.3 Voxel-wise heritability patterns

We extended voxel-level heritability analyses to mean diffusivity (MD) and axial diffusivity (AD) to characterize the shared and modality-specific genetic architecture of white-matter microstructure. Using the same UK Biobank discovery cohort (Phases 1–6; n = 56,313), we performed GWAS on low-dimensional representations for each modality and reconstructed voxel-level SNP heritability estimates.

Consistent with the fractional anisotropy (FA) results, both MD and AD exhibited widespread heritability across the white-matter skeleton after correction for the effective number of voxel-level tests ($P < 0.05/5479.48$). The proportion of significantly heritable voxels was 92.3% for MD and 93.0% for AD, comparable to the 90.6% observed for FA. Across modalities, voxel-level heritability estimates spanned a broad range, with mean values of 15.9% for MD and 14.6% for AD, relative to 16.4% for FA (Supplementary Fig. SX). These results indicate that genetic influences on white-matter microstructure are pervasive and detectable across complementary diffusion measures.

At the anatomical level, heritability patterns for MD and AD recapitulated many of the spatial features observed for FA, with strong signals concentrated in major projection and callosal tracts. For example, the genu of the corpus callosum (GCC) consistently exhibited among the highest and most spatially uniform heritability across all three modalities (FA: 25.1%, MD: 26.2, AD: 22.8%), reflecting strong genetic influences on commissural fiber microstructure. By contrast, several other robustly heritable tracts displayed markedly more heterogeneous patterns. The superior corona radiata (SCR; FA: 20.2%, MD: 17.0%, AD: 17.5%) and external capsule (EC; FA: 20.3%, MD: 14.6%, AD: 14.1%) exhibited the largest within-tract variability across modalities (voxel-level coefficient of variation > 0.39), consistent with their complex crossing-fiber and mixed-fiber architecture. The posterior limb of the internal capsule (PLIC; FA: 25.6%, MD: 11.6%, AD: 14.3%) showed pronounced cross-modality divergence, with FA-based heritability among the highest observed but substantially reduced MD and AD estimates. These results suggest that genetic regulation of directional coherence in this densely packed projection pathway is not fully captured by isotropic diffusion measures.

Heritability estimates were highly reproducible across independent cohorts. Comparing discovery (Phases 1–5) and validation (Phase 7; n = 15,872), voxel-wise correlations of heritability maps were r = 0.75 for MD and r = 0.68 for AD, comparable to the FA correlation of 0.72. At the cluster level, correlations exceeded 0.9 across all modalities, indicating strong stability of spatial genetic architecture (Supplementary Fig. SX).

Together, these results demonstrate that the genetic architecture of white-matter microstructure is largely shared across diffusion modalities, with consistent anatomical organization and high reproducibility, while also revealing modality-specific patterns that likely reflect distinct microstructural sensitivities of FA, MD, and AD.

2.4 Spatial GWAS atlases

Across MD and AD, we identified 238 and 345 genome-wide significant loci, respectively, providing complementary spatial resolution to the 315 FA loci reported in the main text. To characterize the anatomical distribution of genetic effects across diffusion modalities, we constructed voxel-level GWAS atlases for mean diffusivity (MD) and axial diffusivity (AD), complementing the fractional anisotropy (FA) atlas described in the main text. By projecting low-dimensional GWAS summary statistics back into image space, we obtained spatially resolved association maps for each variant, enabling detailed visualization of locus-specific effects across the white-matter skeleton.

Across both MD and AD, genome-wide significant loci exhibited diverse spatial signatures, ranging from highly localized effects confined to specific tracts to more distributed patterns spanning multiple white-matter systems. Similar to FA, many loci demonstrated strong enrichment in major projection and callosal pathways, including the posterior limb of the internal capsule (PLIC), corona radiata, and corpus callosum. However, compared to FA, association patterns in MD and AD were often more spatially diffuse, particularly in association tracts and regions with complex fiber architecture (Fig. SX).

To illustrate these patterns, we examined voxel-level Z-score maps for several representative loci selected based on statistical significance and cross-modality contrast. At locus 5q14.1 (lead SNP: rs115877304), mean diffusivity exhibited a highly focal pattern of association concentrated in the splenium of the corpus callosum (137 discovery voxels), while the same locus showed substantially broader effects in axial diffusivity, with peak effects shifting to the posterior thalamic radiation (1,655 voxels) and extending into the superior longitudinal fasciculus, sagittal stratum, and posterior corona radiata. In fractional anisotropy, the locus spanned all five of these tracts simultaneously, with primary effects in the superior longitudinal fasciculus. These findings illustrate how a single genomic region can produce qualitatively distinct spatial signatures across diffusion modalities, with MD capturing a more focused callosal signal and AD revealing a broader projection-pathway footprint (Fig. SX).

A second locus, 12q23.3 (lead SNP: rs12146713), demonstrated among the most spatially extensive pattern observed across all three modalities. Association signals in FA spanned 12 white-matter tracts (25,013 discovery voxels in the superior corona radiata alone), including bilateral involvement of the corona radiata, corpus callosum, superior longitudinal fasciculus, posterior thalamic radiation, and internal capsule. In MD, the same locus involved 10 tracts with particular enrichment in the genu of the corpus callosum and superior corona radiata, while AD revealed 13 tracts with an additional contribution from the fornix and sagittal stratum not detected in FA. Eight tracts were consistently implicated across all three modalities, indicating a broad shared causal influence on white-matter microstructure at this locus (Fig. SX).

In contrast, some loci displayed marked cross-modality differences in their leading spatial signatures. At 7p22.3, FA showed strong and spatially concentrated associations centered on the superior corona radiata (12,857 discovery voxels) and superior longitudinal fasciculus (3,947 voxels), with a distinct lead SNP (rs798496) relative to both MD (rs1182176) and AD (rs798511). Despite all three modalities implicating

overlapping tract systems, the divergence in lead variants and spatial weighting is consistent with multiple nearby causal variants exerting modality-specific microstructural effects within the same genomic region. Conversely, the locus at 18p11.21 (lead SNP: rs2306811) exhibited pronounced and spatially coherent associations in both MD and AD within the body of the corpus callosum (2,319 and 2,396 discovery voxels, respectively), with no corresponding signal detected in FA. These examples highlight that certain genetic variants preferentially influence specific aspects of tissue microstructure captured by different diffusion measures, and that cross-modality concordance is locus-dependent.

At a global level, spatial GWAS atlases for MD and AD recapitulated the major organizational features observed for FA while revealing additional heterogeneity in association patterns. Projection tracts tended to exhibit strong, spatially coherent signals across all modalities, whereas association tracts showed greater variability in spatial extent and modality specificity, and callosal tracts displayed a mix of highly consistent and locus-specific patterns across modalities.

2.5 Replication of white-matter loci

To evaluate the robustness of the identified loci, we performed replication in an independent held-out cohort (UK Biobank Phase 7; n = 15,872; see §1.1). We assessed two complementary criteria: directional concordance of effect signs, and spatial concordance of voxel-level signal patterns.

Nearly all loci showed directional replication. Of the 315 FA loci identified in the discovery GWAS, 311 (98.7%) were replicated in at least one tract in the validation cohort. Directional replication rates were similarly high for MD (233 of 238 loci, 97.9%) and AD (339 of 345 loci, 98.3%), indicating that effect directions at genome-wide significant loci are highly consistent across independent samples.

Beyond directional concordance, we assessed spatial replication using the voxel-level overlap between discovery and replication association maps. Of the directionally replicated loci, 224 of 311 FA loci (72.0%), 158 of 233 MD loci (67.8%), and 218 of 339 AD loci (64.3%) exhibited nonzero spatial overlap. Defining substantial spatial replication as at least 10% overlap between discovery and replication voxel maps, 207 of 311 FA loci (66.6%), 140 of 233 MD loci (60.1%), and 202 of 339 AD loci (59.6%) met this threshold. FA loci showed modestly higher average spatial overlap (mean 0.43) than MD (mean 0.39) or AD (mean 0.36), consistent with the generally higher heritability of FA relative to diffusivity measures. Across modalities, replicated loci spanned a mean of approximately 3.2–3.4 tracts, and the majority of multi-tract loci reproduced their discovery footprint across multiple tracts in the replication sample.

Among the illustrative loci highlighted in §2.4, spatial replication was particularly strong at the chromosome 12q23.3 locus (rs12146713), which showed high voxel overlap across 12 FA tracts (overlap 0.72), 10 MD tracts (0.71), and 13 AD tracts (0.63), confirming its role as a broadly distributed cross-modality locus. The 5q14.1 locus (rs115877304) replicated across all five of its discovery tracts in FA and AD (overlap 0.21 and 0.02, respectively) and its single MD tract (overlap 0.20), consistent with its modality-shared signal. At the 7p22.3 locus, the FA footprint showed strong replication across eight tracts (overlap 0.64), while the distinct MD and AD lead variants at

this locus also replicated across nine and eleven tracts, respectively (overlap 0.39 and 0.41), supporting modality-specific fine-mapping at a shared genomic region. The 18p11.21 locus (rs2306811) was absent in FA but replicated directionally in both MD and AD across the body of the corpus callosum and adjacent tracts, with modest spatial overlap (MD 0.05, AD 0.003), suggesting that replication voxel distributions in the validation cohort were somewhat attenuated relative to discovery, as expected from reduced sample size.

To contextualize our findings against prior work, we compared identified loci with those reported by Zhao et al. (2021), the largest published GWAS of white-matter tract microstructure to date. The majority of loci identified in the present study were not previously reported: 258 of 315 FA loci (81.9%), 186 of 238 MD loci (78.2%), and 298 of 345 AD loci (86.4%) showed no overlap with Zhao et al. within a $\pm$500 kb window on the same chromosome, with even higher novelty rates when requiring overlap within the same tract (87.0%, 85.3%, and 92.5%, respectively). At the same time, our study recovered the substantial majority of the distinct loci previously identified by Zhao et al.: 57 of their 70 global FA loci (81.4%), 52 of 61 MD loci (85.2%), and 46 of 55 AD loci (83.6%) had a corresponding association in our discovery sample. This high recovery rate, combined with the large number of novel loci, reflects the improved sensitivity of the fPCA-based framework: by representing each tract's diffusion signal through multiple low-dimensional components rather than a single summary statistic, the approach enables reconstruction of voxel-level GWAS signals and detection of spatially focal effects that would otherwise be attenuated. The present study also benefits from a modestly larger discovery cohort (n=56,313 versus n$\approx$33,000 in Zhao et al.). Together, these comparisons indicate that the cross-tract spatial GWAS framework identifies a substantially expanded genetic architecture of white-matter microstructure relative to prior tract-level analyses.

Collectively, the high directional replication rates (>97% across all three modalities) and the substantial spatial concordance at the majority of loci confirm that the identified genetic associations reflect robust, reproducible effects on white-matter microstructure. The modestly lower spatial replication rates relative to directional replication rates reflect the expected attenuation of spatial footprints in the smaller replication sample, rather than failure to replicate the underlying associations (see also Supplementary Fig. SX [spatial replication heatmap]).

2.6 Genetic correlation results

We quantified shared polygenic architecture between fractional anisotropy (FA) and a broad set of neuropsychiatric, neurodegenerative, cognitive, and systemic traits using linkage disequilibrium score regression (LDSC). Location-wise genetic correlations (r_g) revealed distributed, network-structured patterns across the white-matter skeleton, consistent with the results presented in the main text.

Beyond the primary traits highlighted previously, additional phenotypes exhibited spatially structured genetic correlation patterns with FA. For example, schizophrenia and educational attainment showed significant voxel-level correlations concentrated in callosal and frontoparietal association pathways, whereas systemic traits such as C-reactive protein and body mass index demonstrated more diffuse patterns spanning

projection and association tracts. These findings extend the range of traits exhibiting shared genetic architecture with white-matter microstructure beyond canonical neuropsychiatric and cognitive phenotypes.

At the voxel level, significant genetic correlations were generally sparse, with most traits showing associations in only a small fraction of locations after correction for multiple testing. However, these signals were spatially organized rather than random, often aligning with known white-matter systems. Traits related to neurodegeneration and cognition preferentially mapped to commissural and projection pathways, whereas psychiatric and inflammatory traits showed broader involvement of association and callosal tracts.

Genome-wide summary measures further highlighted variability across traits. In addition to the patterns reported in the main text (e.g., Alzheimer's disease and major depressive disorder), traits such as educational attainment and schizophrenia exhibited notable genome-wide correlations with FA, indicating both positive and negative shared genetic influences on white-matter microstructure.

At the tract level, aggregation of voxel-wise signals identified numerous significant tract-trait pairs involving a broad set of tracts and traits. In addition to the major associations described in the main text, educational attainment showed enrichment in the posterior limb of the internal capsule, while schizophrenia and bipolar disorder exhibited stronger associations in the corpus callosum and superior longitudinal fasciculus. Directional agreement within tracts remained high, indicating coherent genetic effects along anatomically defined pathways.

2.7 Mendelian randomization results

We further characterized directional relationships between fractional anisotropy (FA) and complex traits using two-sample Mendelian randomization (MR), extending the primary results described in the main text. Analyses were conducted under the same conservative multi-estimator framework, requiring FDR-corrected inverse-variance weighted significance and concordance across multiple MR methods.

In addition to the trait associations highlighted previously, several additional phenotypes exhibited robust directional effects on white-matter microstructure. Educational attainment, schizophrenia, and inflammatory traits demonstrated significant tract-level associations, with effects localized to major projection, commissural, and association pathways. These results expand the range of traits with evidence for putative causal influence on FA beyond those emphasized in the main text.

Consistent with the primary findings, directional effects were strongly network-dependent. Neurodegenerative and cognitive traits tended to exhibit concentrated effects within projection pathways, whereas psychiatric and systemic traits showed broader involvement of association and callosal tracts. In several cases, traits with modest or diffuse genetic correlation profiles nonetheless demonstrated localized directional effects, suggesting that causal influence may be restricted to specific anatomical systems even when genome-wide overlap is limited.

We also observed heterogeneity in the strength and consistency of MR signals across traits. Some traits exhibited robust effects supported by all major estimators, whereas others showed weaker or less consistent evidence despite detectable genetic

correlation. These differences highlight the importance of multi-method validation in distinguishing robust causal signals from pleiotropic associations.

Across all analyses, significant tract-trait pairs were concentrated in large-scale white-matter systems, including the superior longitudinal fasciculus, superior corona radiata, sagittal stratum, and internal capsule, reinforcing the role of these pathways as key substrates linking complex traits to brain microstructure.

2.8 Multi-omics associations and cross-omics spatial concordance

We extended the multi-omics analyses described in the main text by examining additional molecular features and characterizing cross-omics spatial structure of association patterns. Consistent with primary findings, metabolomic features exhibited the strongest and most spatially extensive associations with fractional anisotropy (FA), followed by proteomic features, whereas clinical biomarkers showed minimal or no spatially structured effects after correction.

Beyond the features highlighted in the main text, several additional metabolites and proteins demonstrated significant associations with FA. These included lipid-related metabolites, inflammatory proteins, and markers of neuronal and vascular function, with effects ranging from localized signals in commissural pathways to more distributed patterns across association and projection tracts. These results indicate that molecular influences on white-matter microstructure extend beyond the most prominent signals and involve a broad set of biological pathways.

To quantify cross-omics spatial structure, we computed pairwise Pearson correlations of voxel-level Z-score maps across all molecular features. Consistent with the main text, clustering occurred primarily by omics type, with metabolomic features showing the highest within-type concordance, followed by proteomic features, whereas cross-type correlations were generally lower.

Despite low phenotypic correlations between many molecular features, spatial association patterns were often highly concordant. Conversely, some phenotypically correlated features showed limited spatial overlap, indicating that shared biological effects are not always captured by marginal feature correlations alone.

We further quantified overlap of statistically significant regions using Dice coefficients after FDR correction, identifying numerous feature pairs with substantial spatial overlap. Several of the strongest overlaps occurred across omics layers, including metabolite-gene and metabolite-protein pairs, indicating convergence of genetic and molecular influences on specific white-matter pathways.

Together, these findings demonstrate that multi-omics associations with white-matter microstructure are both modality-dependent and spatially structured, with convergent effects across distinct molecular layers on shared anatomical systems.

2.9 Biological enrichment and networks

We extended the biological enrichment and network analyses described in the main text by summarizing the full set of mapped genes and enriched pathways across diffusion modalities. Gene mapping of genome-wide significant loci identified hundreds of genes across FA, MD, and AD, including a subset unique to individual modalities.

Gene-set enrichment analysis confirmed overrepresentation of pathways related to neurogenesis, cytoskeletal organization, synaptic signaling, myelination, and extracellular matrix structure, consistent with the primary findings. Additional enriched terms included axon guidance, cell adhesion, and immune signaling, further supporting involvement of diverse biological processes underlying white-matter microstructure. Modality-specific differences were modest, with FA-associated genes showing relatively stronger enrichment in neuronal development and axonal organization pathways, whereas MD and AD signals were more prominent in extracellular and structural processes.

Protein-protein interaction networks exhibited significant enrichment for interactions and revealed multiple connected components corresponding to functional modules involved in signaling, cytoskeletal regulation, and extracellular communication.

Integration of genetic and molecular features through gene-metabolite network analysis identified interconnected modules linking mapped genes to circulating metabolites. These networks demonstrated extensive cross-layer connectivity, supporting convergence of genetic and metabolic influences on shared white-matter pathways.

Overall, these analyses provide convergent biological evidence that genetic influences on white-matter microstructure act through coordinated pathways involved in neurodevelopment, axonal maintenance, and systemic metabolic regulation.

References

Bookheimer SY, Salat DH, Terpstra M, et al (2019) The lifespan human connectome project in aging: an overview. Neuroimage 185:335–348

Bycroft C, Freeman C, Petkova D, et al (2018) The uk biobank resource with deep phenotyping and genomic data. Nature 562(7726):203–209

Casey BJ, Cannonier T, Conley MI, et al (2018) The adolescent brain cognitive development (abcd) study: imaging acquisition across 21 sites. Developmental cognitive neuroscience 32:43–54

Chang CC, Chow CC, Tellier LC, et al (2015) Second-generation plink: rising to the challenge of larger and richer datasets. Gigascience 4(1):s13742–015

Chen Z, Boehnke M, Wen X, et al (2021) Revisiting the genome-wide significance threshold for common variant gwas. G3 11(2):jkaa056

Demontis D, Walters GB, Athanasiadis G, et al (2023) Genome-wide analyses of adhd identify 27 risk loci, refine the genetic architecture and implicate several cognitive domains. Nature genetics 55(2):198–208

Elliott LT, Sharp K, Alfaro-Almagro F, et al (2018) Genome-wide association studies of brain imaging phenotypes in uk biobank. Nature 562(7726):210–216

Fan J (2018) Local polynomial modelling and its applications: monographs on statistics and applied probability 66. Routledge

Giambartolomei C, Vukcevic D, Schadt EE, et al (2014) Bayesian test for colocalisation between pairs of genetic association studies using summary statistics. PLoS genetics 10(5):e1004383

Grove J, Ripke S, Als TD, et al (2019) Identification of common genetic risk variants for autism spectrum disorder. Nature genetics 51(3):431–444

Hemani G, Zheng J, Elsworth B, et al (2018) The mr-base platform supports systematic causal inference across the human phenome. elife 7:e34408

Hoerl AE, Kennard RW (1970) Ridge regression: Biased estimation for nonorthogonal problems. Technometrics 12(1):55–67

Jiang Z, Stein J, Li T, et al (2026) Computation and resource efficient genome-wide association analysis for large-scale imaging studies. Nature Communications

Kolberg L, Raudvere U, Kuzmin I, et al (2023) g: Profiler—interoperable web service for functional enrichment analysis and gene identifier mapping (2023 update). Nucleic acids research 51(W1):W207–W212

Lee JJ, Wedow R, Okbay A, et al (2018) Gene discovery and polygenic prediction from a genome-wide association study of educational attainment in 1.1 million individuals. Nature genetics 50(8):1112–1121

Li T, Wang X, Cole M, et al (2026) Connectome-based spatial statistics enabling large-scale population analyses of human connectome across cohorts Under review

Liu Y, Chen S, Li Z, et al (2019) Acat: a fast and powerful p value combination method for rare-variant analysis in sequencing studies. The American Journal of Human Genetics 104(3):410–421

Loh PR, Tucker G, Bulik-Sullivan BK, et al (2015) Efficient bayesian mixed-model analysis increases association power in large cohorts. Nature genetics 47(3):284–290

Marees AT, De Kluiver H, Stringer S, et al (2018) A tutorial on conducting genome-wide association studies: Quality control and statistical analysis. International journal of methods in psychiatric research 27(2):e1608

Mazziotta J, Toga A, Evans A, et al (2001) A probabilistic atlas and reference system for the human brain: International consortium for brain mapping (icbm). Philosophical Transactions of the Royal Society of London Series B: Biological Sciences 356(1412):1293–1322

Miller KL, Alfaro-Almagro F, Bangerter NK, et al (2016) Multimodal population brain imaging in the uk biobank prospective epidemiological study. Nature neuroscience 19(11):1523–1536

Mullins N, Forstner AJ, O'Connell KS, et al (2021) Genome-wide association study of over 40,000 bipolar disorder cases provides new insights into the underlying biology. Nature genetics 53(6):817

Nagel M, Jansen PR, Stringer S, et al (2018) Meta-analysis of genome-wide association studies for neuroticism in 449,484 individuals identifies novel genetic loci and pathways. Nature genetics 50(7):920–927

Oishi K, Zilles K, Amunts K, et al (2008) Human brain white matter atlas: identification and assignment of common anatomical structures in superficial white matter. Neuroimage 43(3):447–457

Pang Z, Chong J, Zhou G, et al (2021) Metaboanalyst 5.0: narrowing the gap between raw spectra and functional insights. Nucleic acids research 49(W1):W388–W396

Purcell S, Neale B, Todd-Brown K, et al (2007) Plink: a tool set for whole-genome association and population-based linkage analyses. The American journal of human genetics 81(3):559–575

Savage JE, Jansen PR, Stringer S, et al (2018) Genome-wide association meta-analysis in 269,867 individuals identifies new genetic and functional links to intelligence. Nature genetics 50(7):912–919

Shang HL (2014) A survey of functional principal component analysis. AStA Advances in Statistical Analysis 98(2):121–142

Stein JL, Hua X, Lee S, et al (2010) Voxelwise genome-wide association study (vgwas). neuroimage 53(3):1160–1174

Sudlow C, Gallacher J, Allen N, et al (2015) Uk biobank: an open access resource for identifying the causes of a wide range of complex diseases of middle and old age. PLoS medicine 12(3):e1001779

Sun BB, Chiou J, Traylor M, et al (2023) Plasma proteomic associations with genetics and health in the uk biobank. Nature 622(7982):329–338

Szklarczyk D, Gable AL, Lyon D, et al (2019) String v11: protein–protein association networks with increased coverage, supporting functional discovery in genome-wide experimental datasets. Nucleic acids research 47(D1):D607–D613

Trubetskoy V, Pardiñas AF, Qi T, et al (2022) Mapping genomic loci implicates genes and synaptic biology in schizophrenia. Nature 604(7906):502–508

Turner S, Armstrong LL, Bradford Y, et al (2011) Quality control procedures for genome-wide association studies. Current protocols in human genetics 68(1):1–19

Watanabe K, Taskesen E, Van Bochoven A, et al (2017) Functional mapping and annotation of genetic associations with fuma. Nature communications 8(1):1826

Watanabe K, Jansen PR, Savage JE, et al (2022) Genome-wide meta-analysis of insomnia prioritizes genes associated with metabolic and psychiatric pathways. Nature genetics 54(8):1125–1132

Wightman DP, Jansen IE, Savage JE, et al (2021) A genome-wide association study with 1,126,563 individuals identifies new risk loci for alzheimer's disease. Nature genetics 53(9):1276–1282

Wray NR, Ripke S, Mattheisen M, et al (2018) Genome-wide association analyses identify 44 risk variants and refine the genetic architecture of major depression. Nature genetics 50(5):668–681

Zhao B, Li T, Yang Y, et al (2021) Common genetic variation influencing human white matter microstructure. Science 372(6548):eabf3736

Supplementary Figures

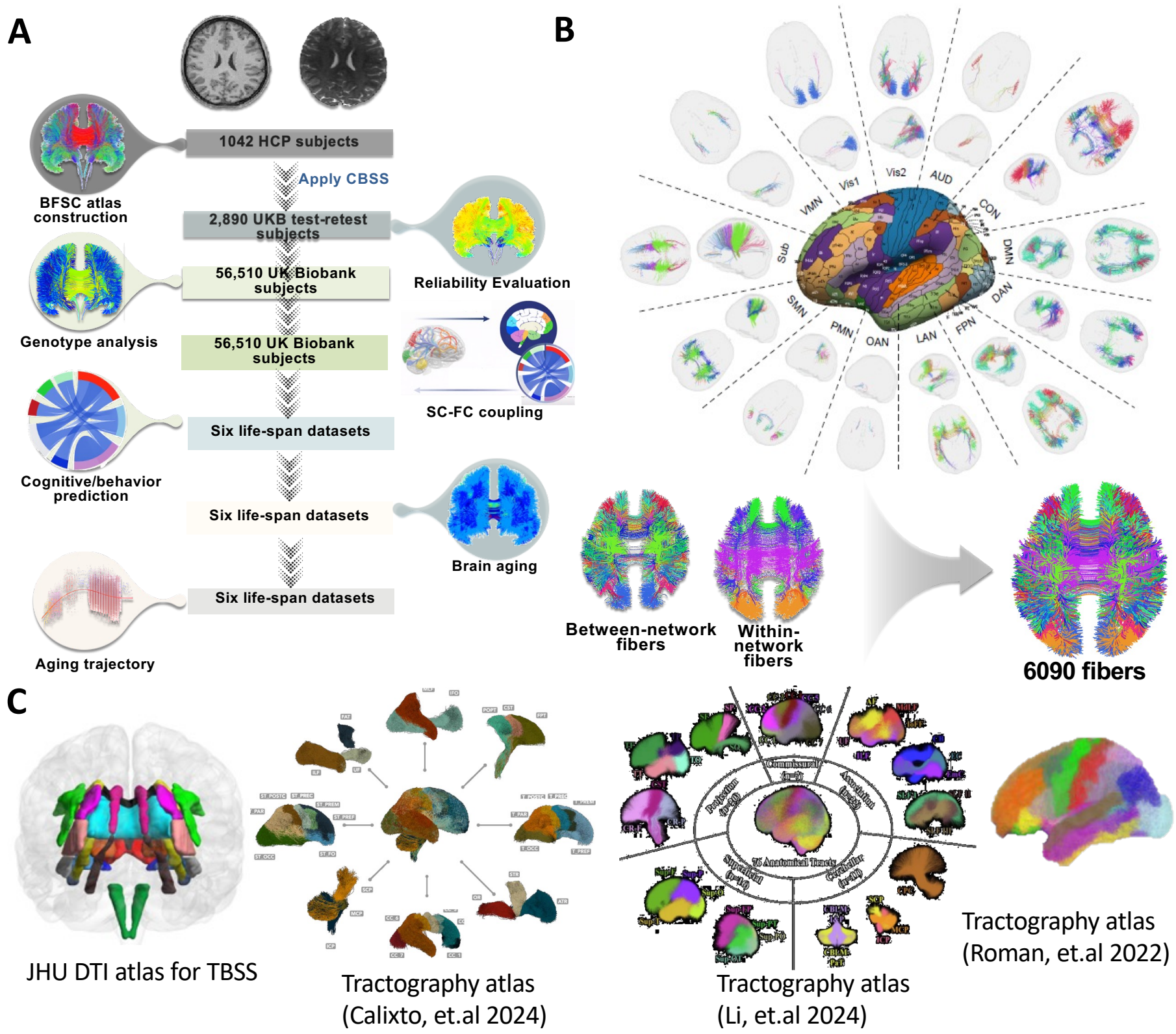

Fig. 1 CBSS white-matter atlas used for fiber-level analyses. (A) Overview of the connectivity-based spatial statistics (CBSS) framework and related neuroimaging applications. CBSS enables population-level analysis of white-matter microstructure using diffusion MRI–derived features and has been applied to diverse settings including brain-age prediction, genetic analysis, brain–behavior associations, and disease studies. **(B)** Construction of the CBSS fiber atlas used in this study. Diffusion tensor imaging (DTI) data are projected onto a white-matter skeleton using tract-based spatial statistics (TBSS), after which spatially contiguous fiber segments are identified and aggregated across subjects. The resulting atlas partitions the white-matter skeleton into thousands of anatomically coherent fiber clusters (6,090 fibers), providing a high-resolution representation of white-matter pathways for voxel-level genetic analyses. The atlas integrates information from existing tractography atlases and enables fine-scale investigation of white-matter microstructure across the brain. **(C)** Reference white-matter atlases used during preprocessing and anatomical alignment, including commonly used tractography and diffusion MRI atlases that facilitate consistent mapping of white-matter structures across subjects and studies.

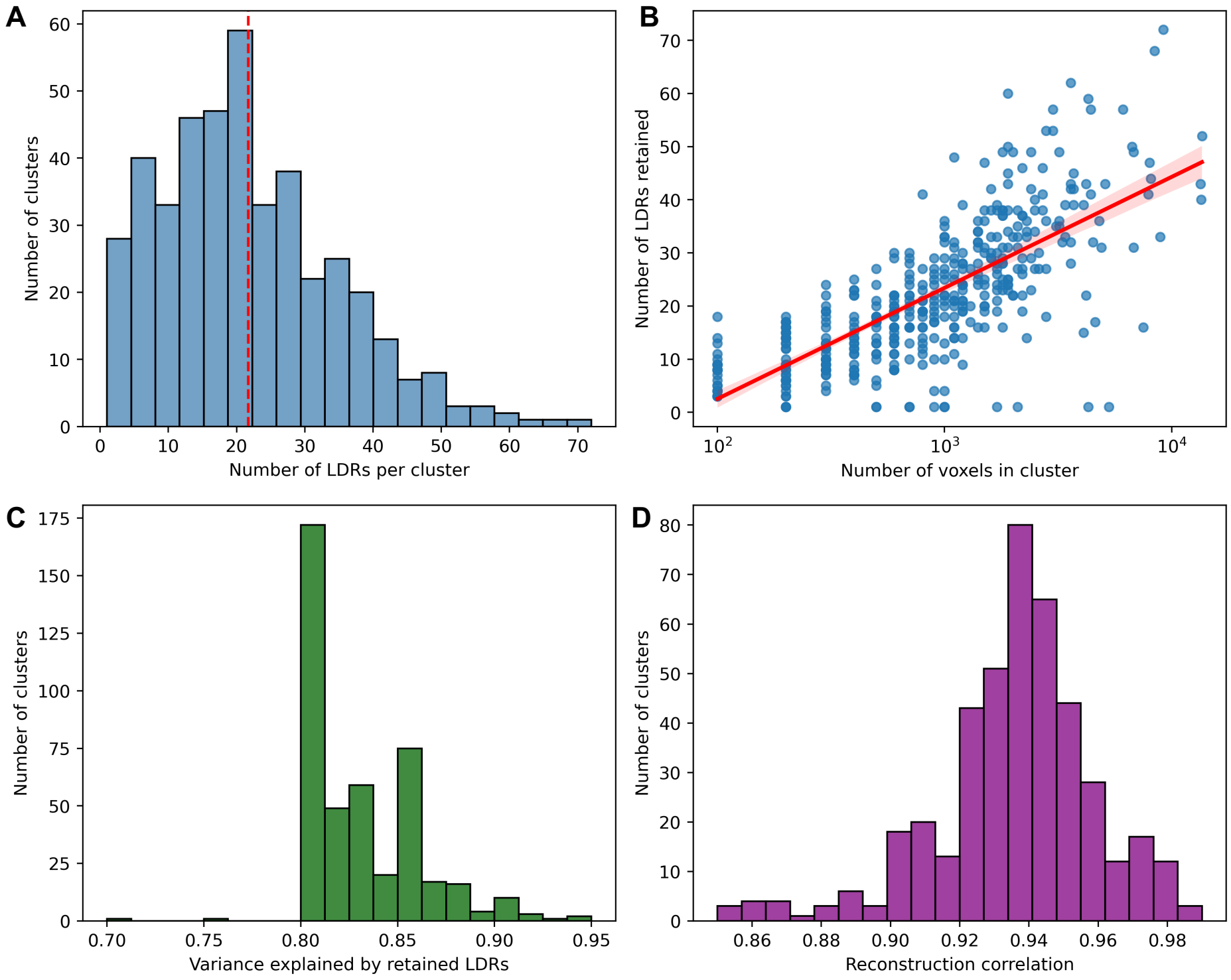

Fig. 2 Properties of low-dimensional representations (LDRs) used to model fiber-level diffusion MRI signals. (A) Distribution of the number of LDRs retained per fiber cluster. The dashed red line indicates the median number of components across clusters. **(B)** Relationship between the number of voxels in a fiber cluster and the number of LDRs retained. Each point represents a fiber cluster, and the red line shows the fitted linear trend. Larger clusters generally require more components to capture spatial variation in diffusion signals. **(C)** Distribution of the proportion of variance explained by the retained LDRs across fiber clusters. Most clusters retain components explaining approximately 80–90% of the total variance. **(D)** Distribution of reconstruction correlations between the original voxel-level diffusion signals and those reconstructed from the retained LDRs, demonstrating high fidelity of the low-dimensional representation across clusters.

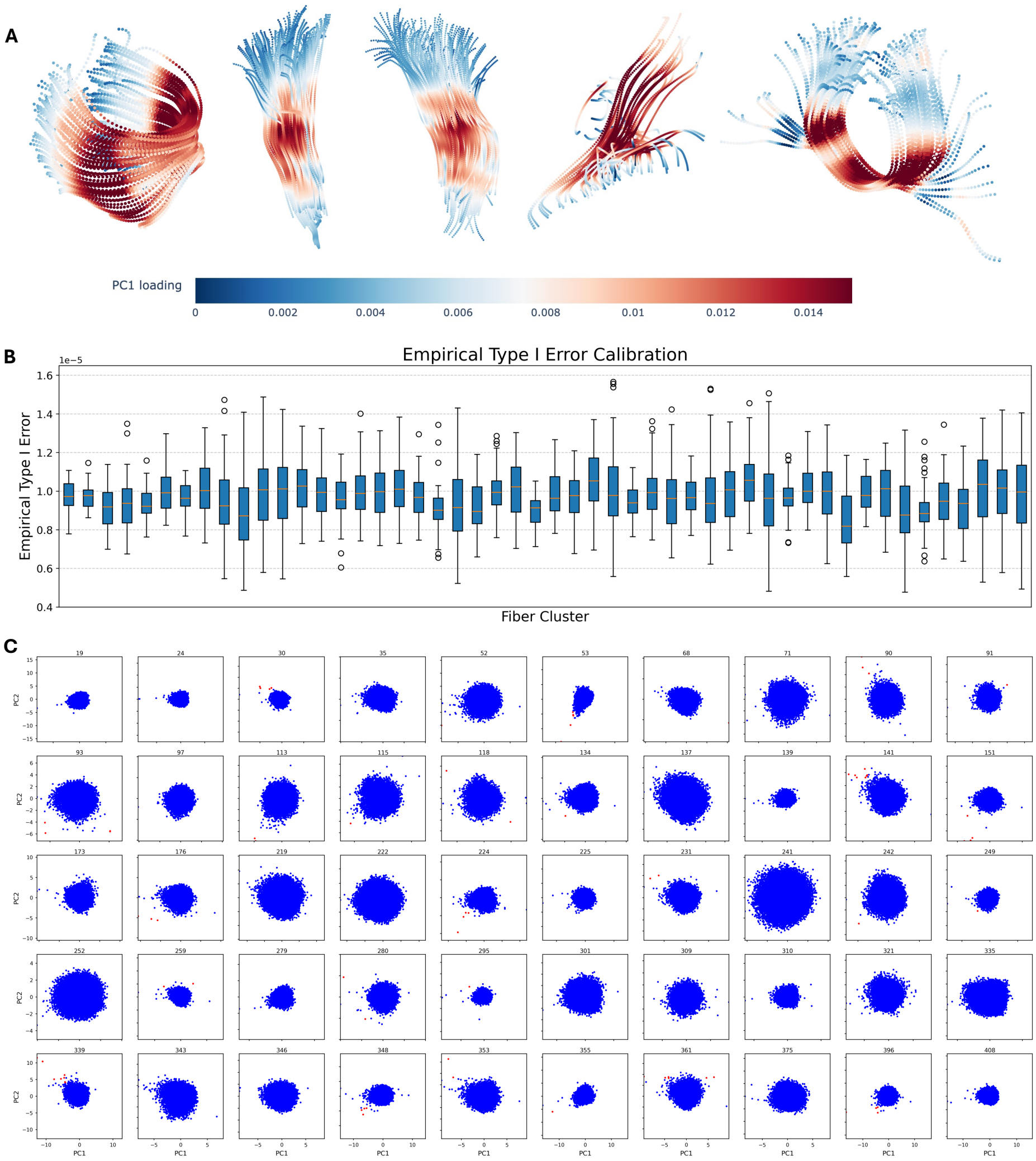

Fig. 3 Quality control and calibration of voxel-level GWAS across fiber clusters. (A) Spatial maps of the first principal component (PC) loadings across voxels for five representative fiber clusters, illustrating the spatial patterns captured by the low-dimensional representation of diffusion signals. **(B)** Empirical Type I error calibration across 50 randomly selected fiber clusters. Each boxplot summarizes the distribution of empirical Type I error estimates obtained from null simulations, normalized by the expected error rate. **(C)** Scatterplots of the top two principal components (PC1 and PC2) for the same 50 randomly selected fiber clusters shown in panel B, illustrating the distribution of LDR scores and potential outliers across clusters.

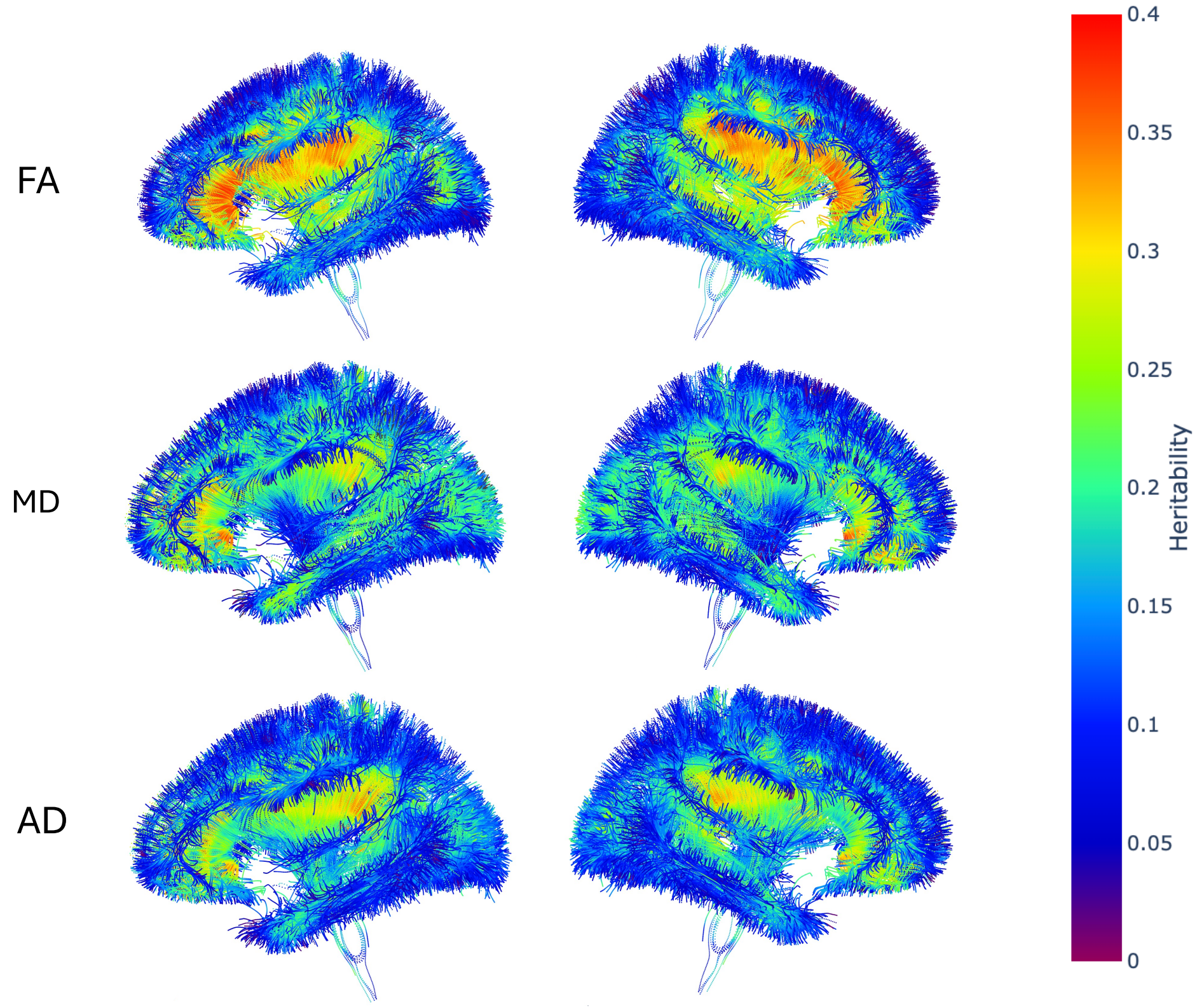

Fig. 4 Voxel-wise heritability of white-matter microstructure across diffusion MRI measures. Voxel-level SNP heritability estimates across the white-matter skeleton for three diffusion MRI measures—fractional anisotropy (FA), mean diffusivity (MD), and axial diffusivity (AD)—illustrating broadly distributed and modality-specific patterns of genetic influence on white-matter microstructure.

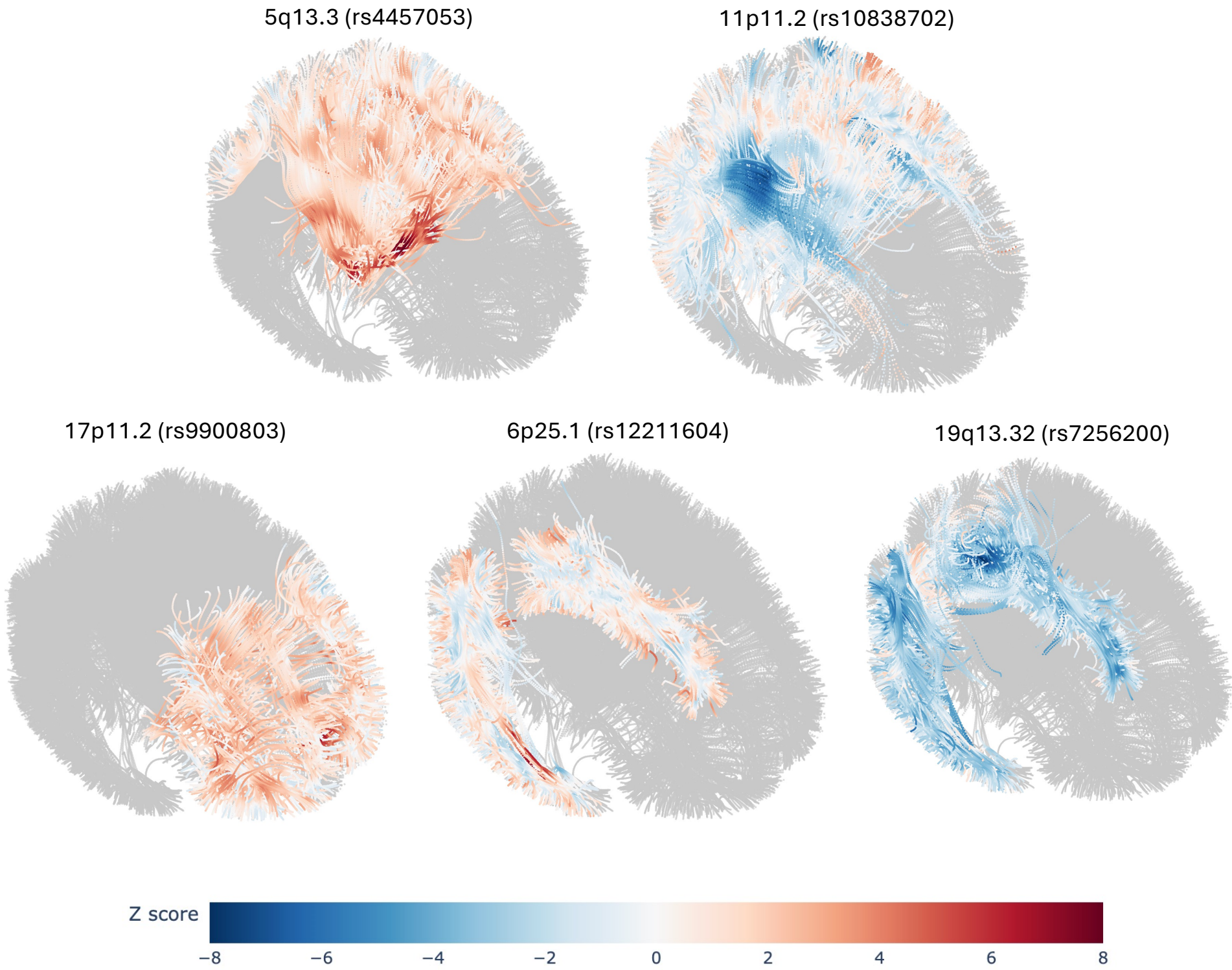

Fig. 5 Spatial atlases of voxel-level genetic effects for representative white-matter loci. Voxel-wise GWAS Z-score maps are shown for five loci associated with white-matter microstructure. Colors indicate SNP effect Z-scores (display range -8 to 8). Each panel shows the spatial distribution of genetic effects across fiber pathways for the lead SNP at the locus. Tracts listed below correspond to fiber pathways in which discovery signals were detected, along with the proportion of overlap observed in replication data. **(A)** 5q13.3 (rs4457053), previously associated with type 2 diabetes, shows effects across projection pathways including the cerebral peduncle (CP), posterior corona radiata (PCR), posterior limb of the internal capsule (PLIC), and superior corona radiata (SCR), with 90.3% overlap in replication. **(B)** 11p11.2 (rs10838702), associated with Alzheimer's disease, exhibits effects in superior corona radiata (SCR) and superior longitudinal fasciculus (SLF), with 80.2% replication overlap. **(C)** 17p11.2 (rs9900803), associated with stroke, shows focal effects in the anterior corona radiata (ACR), with complete replication overlap (100%). **(D)** 6p25.1 (rs12211604), associated with glomerular filtration rate, shows effects in the posterior thalamic radiation (PTR) and sagittal stratum (SS), with complete replication overlap (100%). **(E)** 19q13.32 (rs7256200), associated with coronary atherosclerosis, exhibits effects in posterior thalamic radiation (PTR), splenium of the corpus callosum (SCC), and sagittal stratum (SS), with 63.1% replication overlap.

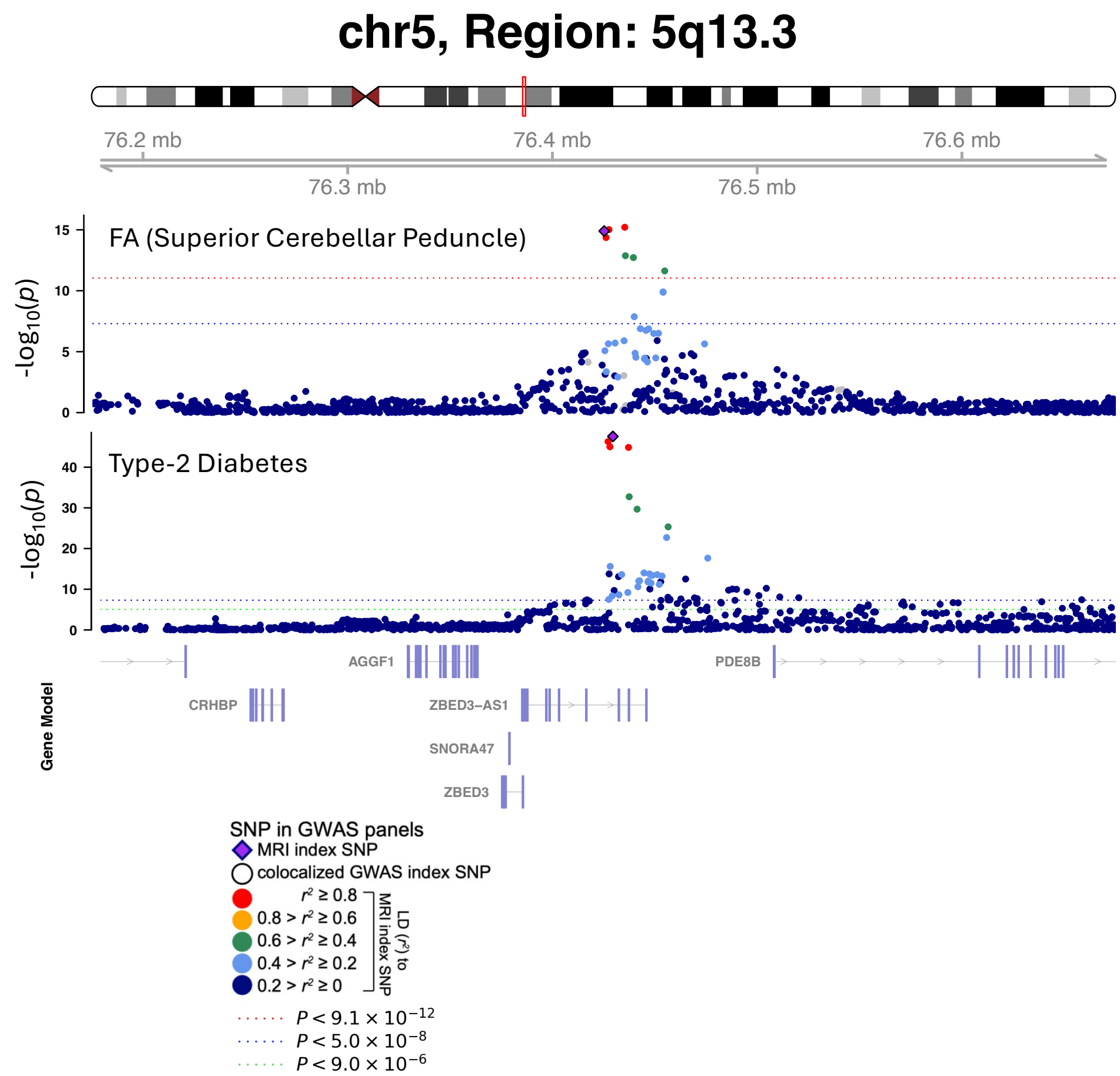

Fig. 6 Colocalization of white-matter microstructure loci with complex traits and diseases. In 5q13.3, we observed evidence of colocalization between white-matter microstructure (FA in SCP, index variant rs4457053) and Type-2 Diabetes (index variant rs6878122). Bayesian colocalization analysis indicated a high posterior probability for a shared causal variant (PP.H4.abf = 0.997).

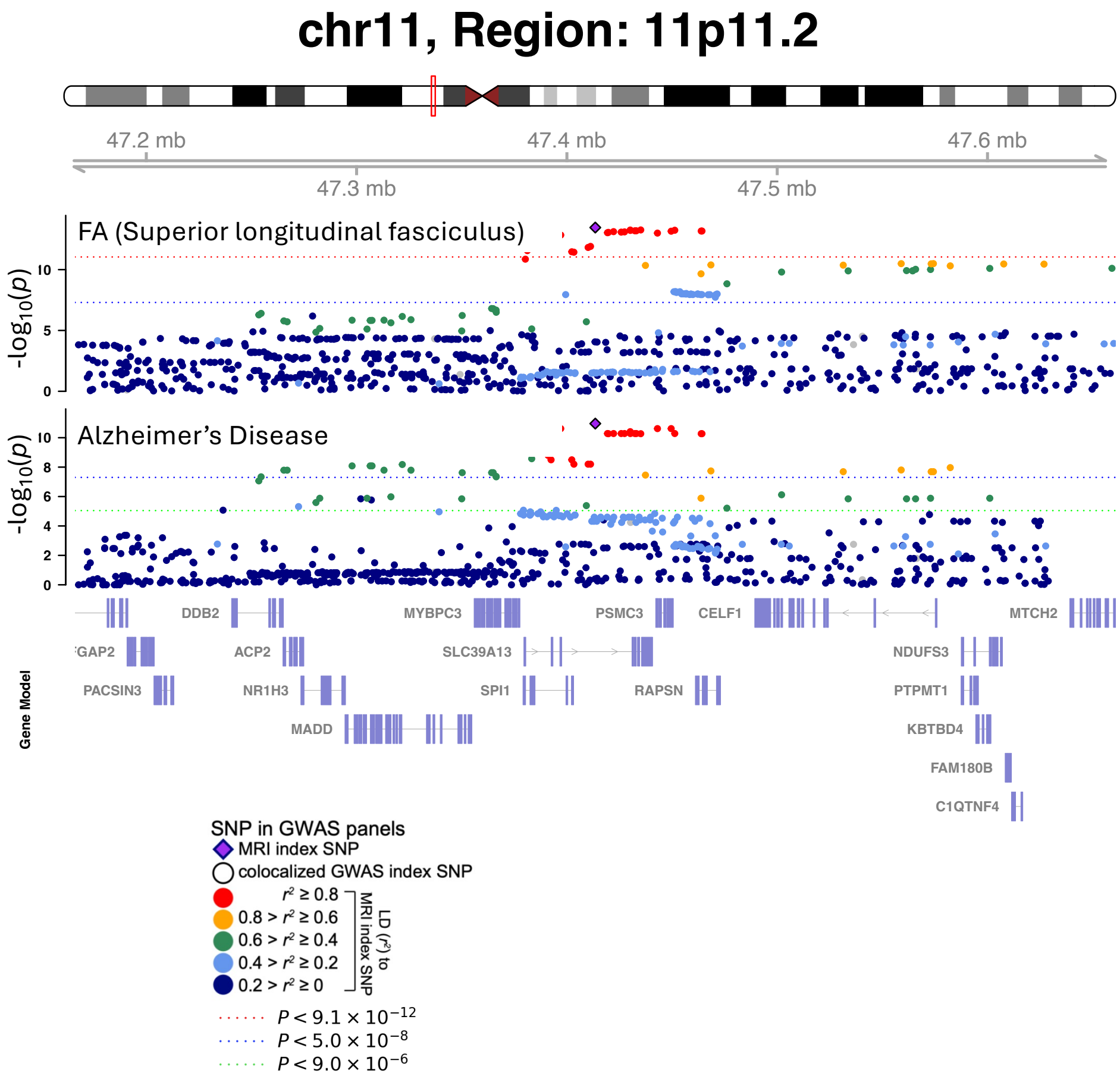

Fig. 7 Colocalization of white-matter microstructure loci with complex traits and diseases. In 11p11.2, we observed evidence of colocalization between white-matter microstructure (FA in SLF) and Alzheimer's Disease (shared index variant rs10838702). Bayesian colocalization analysis indicated a high posterior probability for a shared causal variant (PP.H4.abf = 0.982).

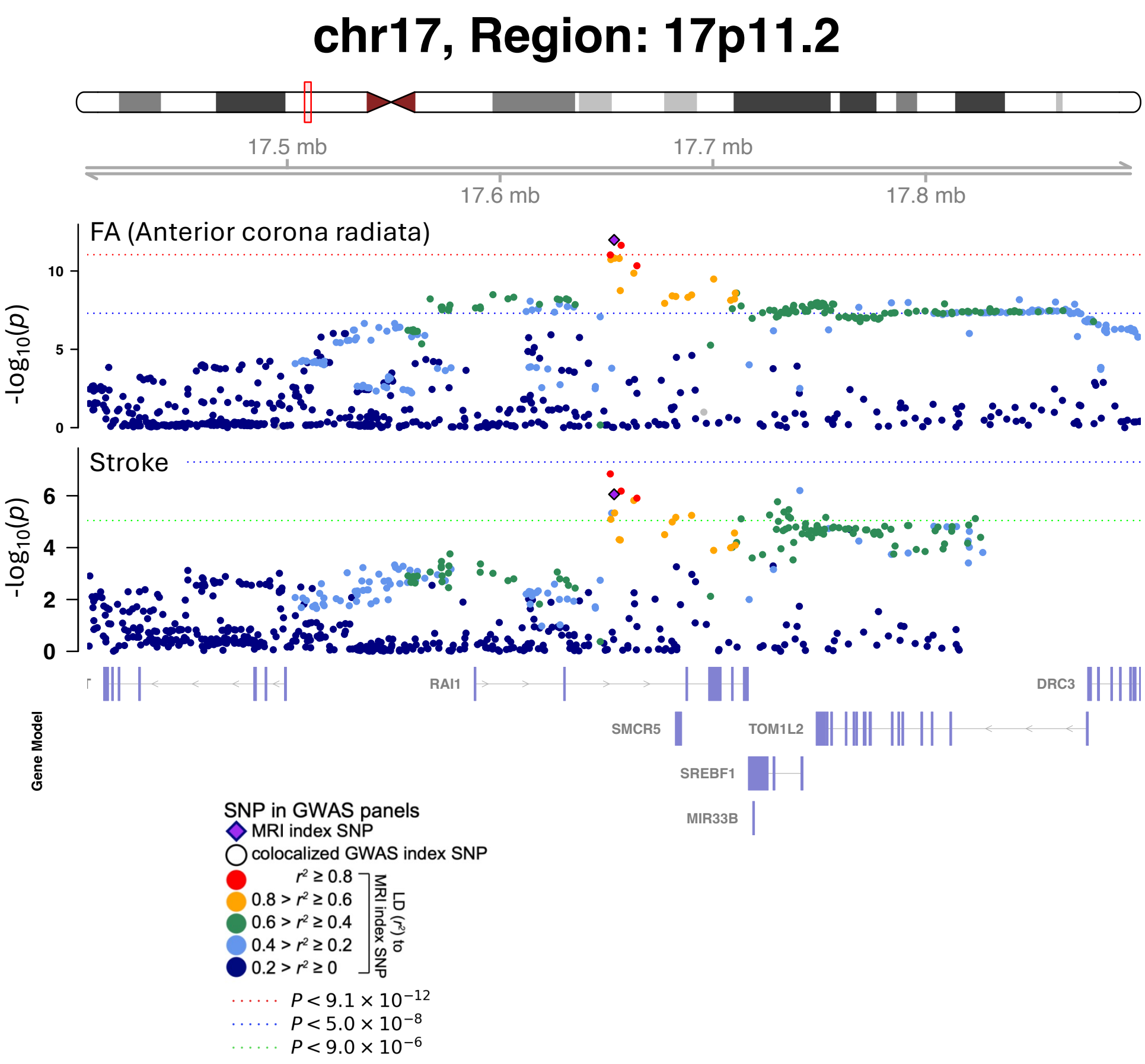

Fig. 8 Colocalization of white-matter microstructure loci with complex traits and diseases. In 17p11.2, we observed evidence of colocalization between white-matter microstructure (FA in ACR) and stroke (shared index variant rs9900803). Bayesian colocalization analysis indicated a high posterior probability for a shared causal variant (PP.H4.abf = 0.986).

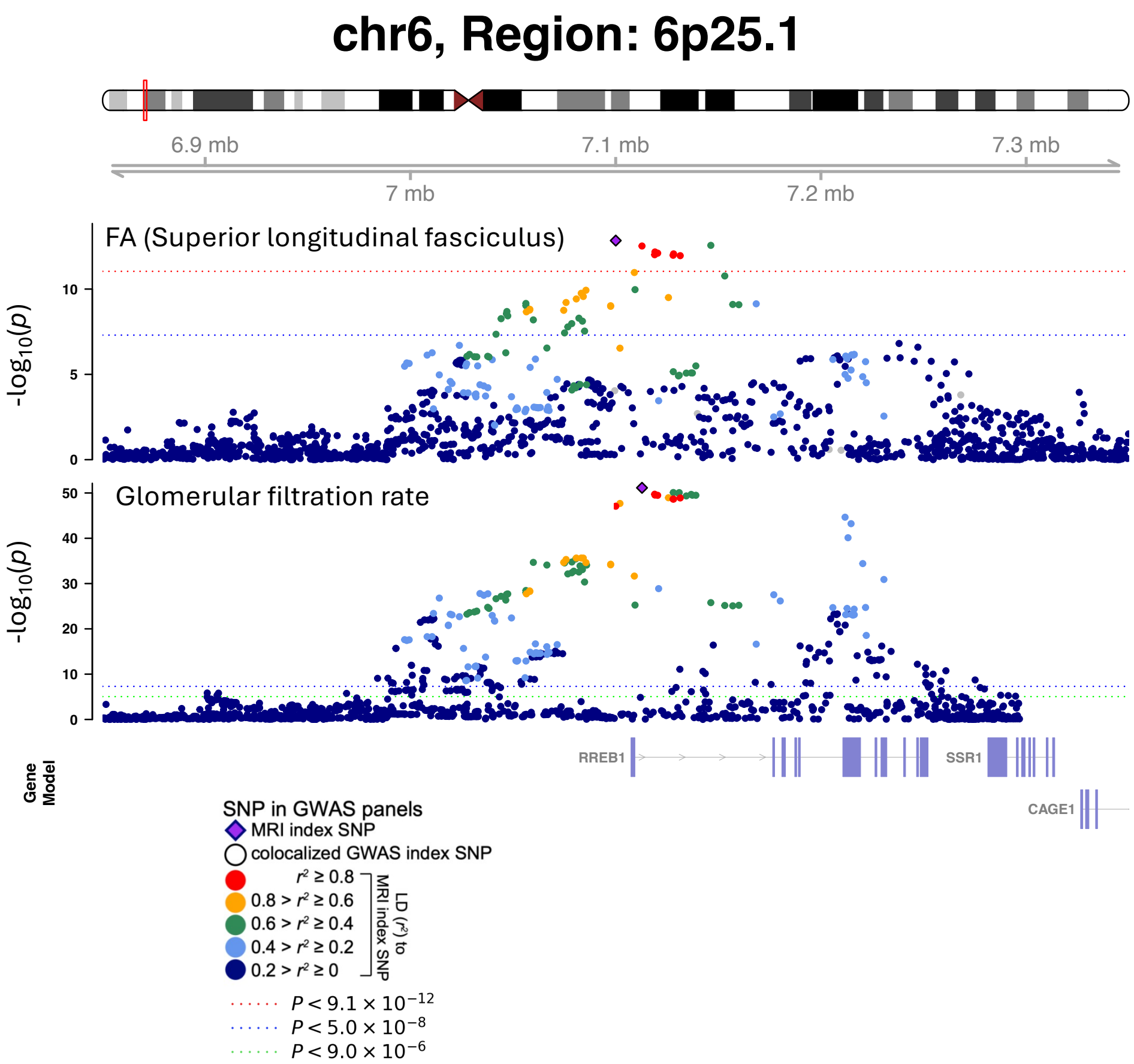

Fig. 9 Colocalization of white-matter microstructure loci with complex traits and diseases. In 6p25.1, we observed evidence of colocalization between white-matter microstructure (FA in SLF, index variant rs12211604) and glomerular filtration rate (index variant rs6925389). Bayesian colocalization analysis indicated a high posterior probability for a shared causal variant (PP.H4.abf = 0.992).

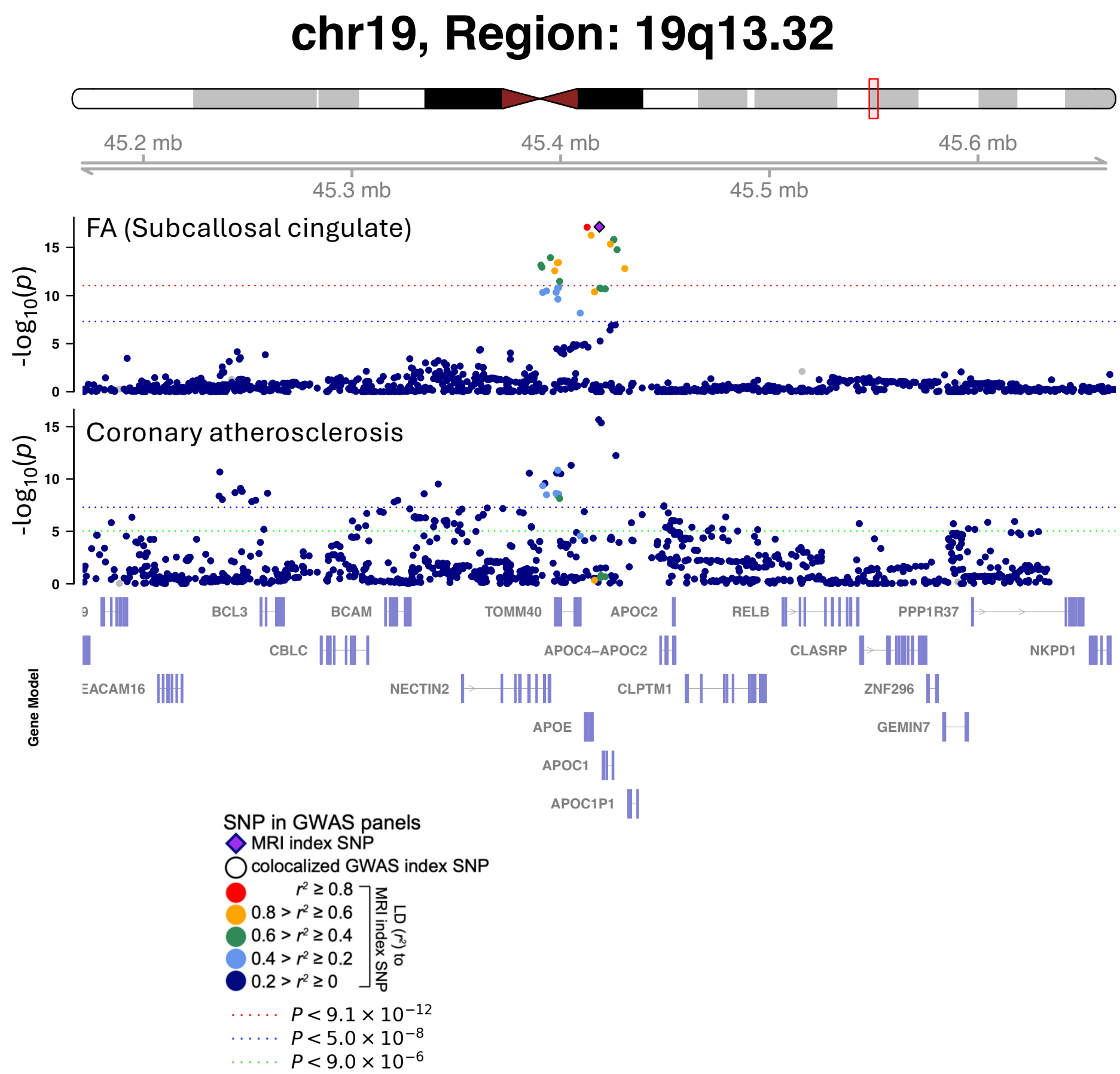

Fig. 10 Colocalization of white-matter microstructure loci with complex traits and diseases. In 19q13.32, we observed evidence of colocalization between white-matter microstructure (FA in SCC, index variant rs7256200) and coronary atherosclerosis (index variant rs12972970). Bayesian colocalization analysis indicated a high posterior probability for a shared causal variant (PP.H4.abf = 0.995).

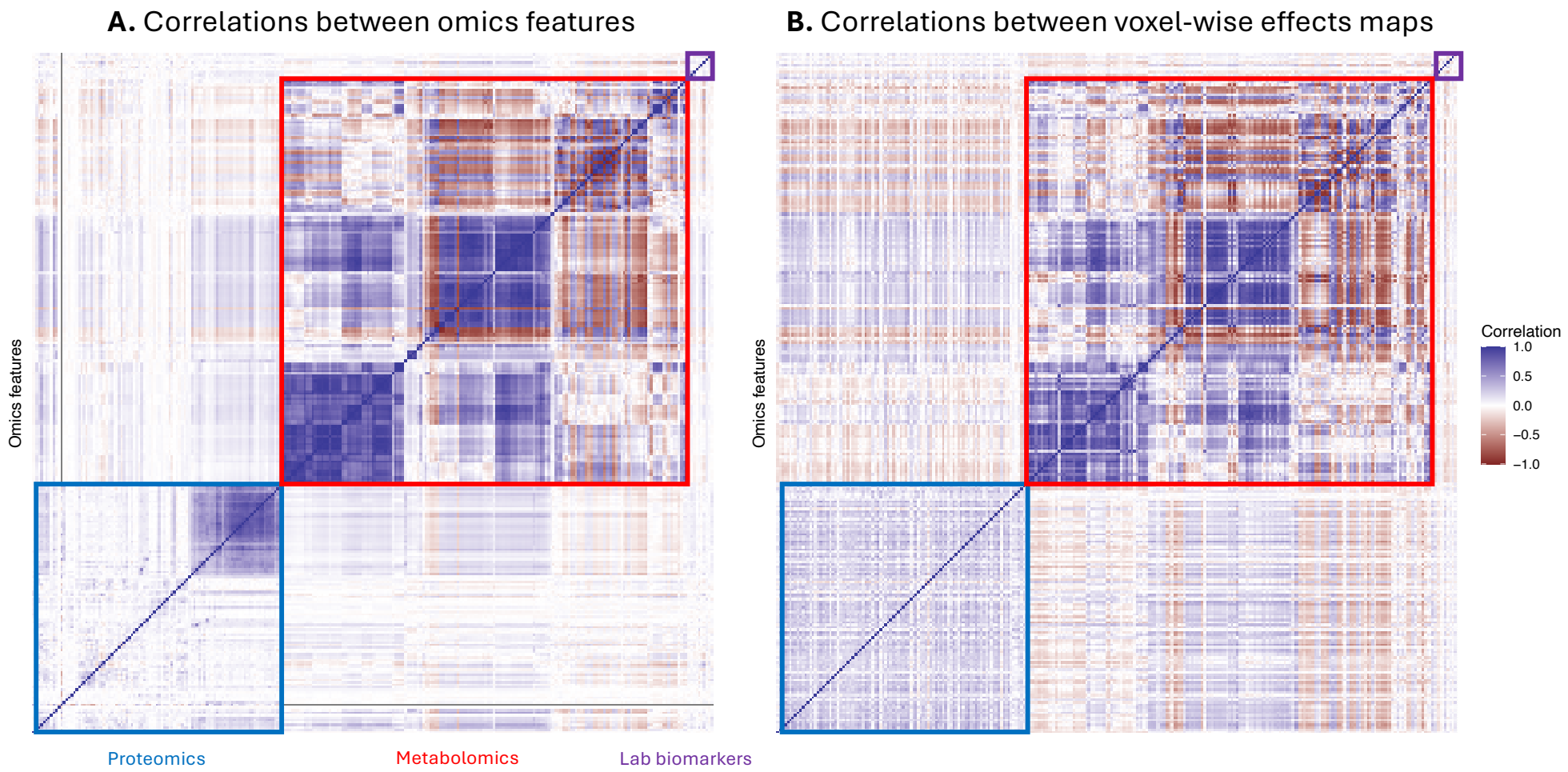

Fig. 11 Phenotypic and effect map correlations across multi-omics features. Heatmaps show pairwise correlations across proteomic, metabolomic, and clinical biomarker features associated with white-matter microstructure. Panel (A) displays phenotypic correlations between features measured in the same individuals. Panel (B) shows correlations between voxel-wise omics effect maps, reflecting similarity in the spatial patterns of omics associations across white-matter fiber pathways. Features are grouped by omics category (proteomic, metabolomic, biomarker), indicated by colored annotation boxes along the axes. Within each category, features are hierarchically clustered based on phenotypic correlations. Color scale indicates Pearson correlation coefficients (r), ranging from -1 to 1.

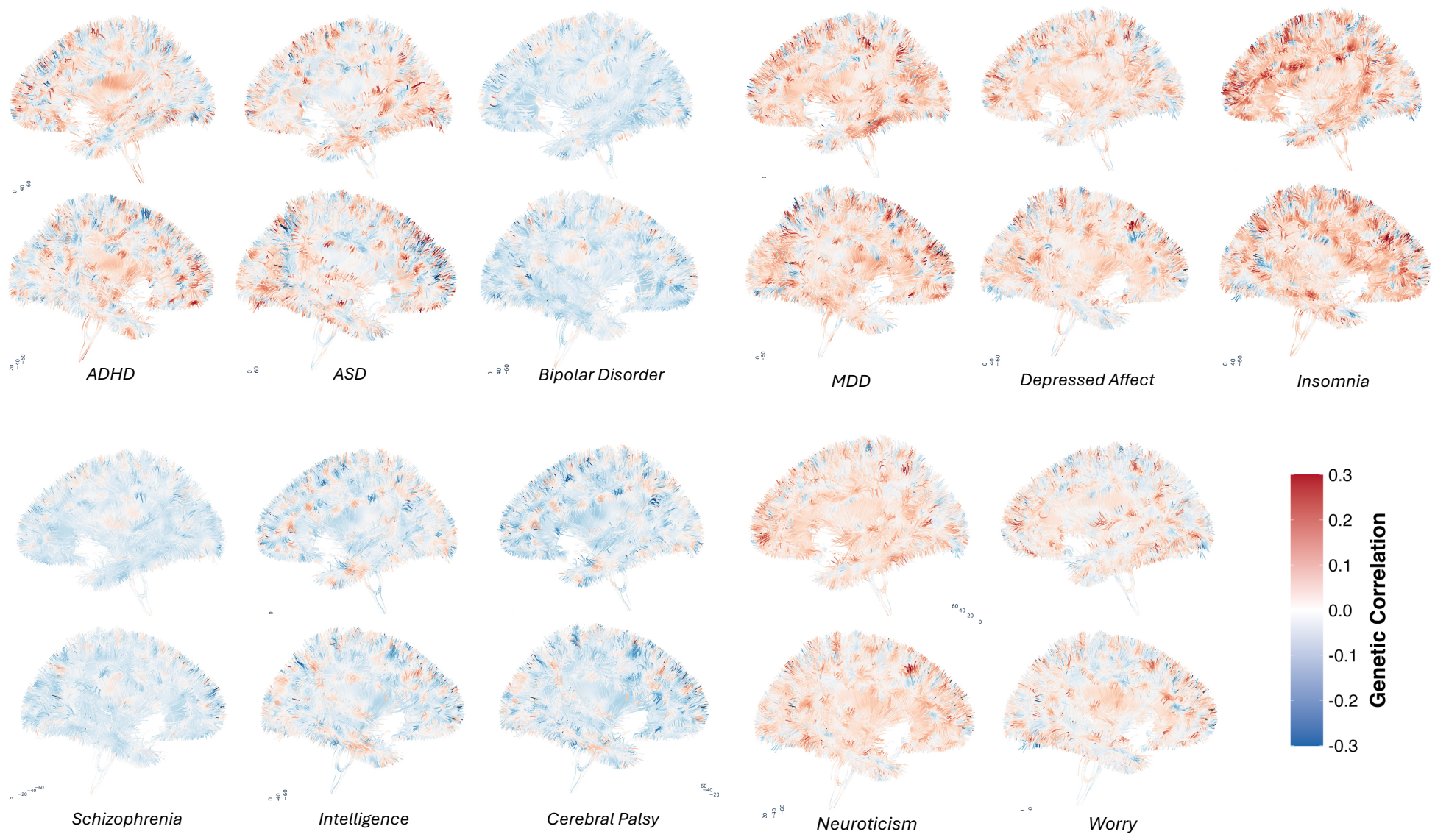

Fig. 12 Fiber-level genetic correlations between white-matter microstructure and brain-related traits. Spatial maps show estimated genetic correlations between voxel-wise fractional anisotropy and 11 brain-related traits, including ADHD, autism spectrum disorder, bipolar disorder, major depressive disorder, depressed affect, insomnia, schizophrenia, intelligence, cerebral palsy, neuroticism, and worry. Correlations were estimated at the fiber level across the white-matter skeleton, with colors indicating the magnitude and direction of genetic correlation (red: positive; blue: negative).

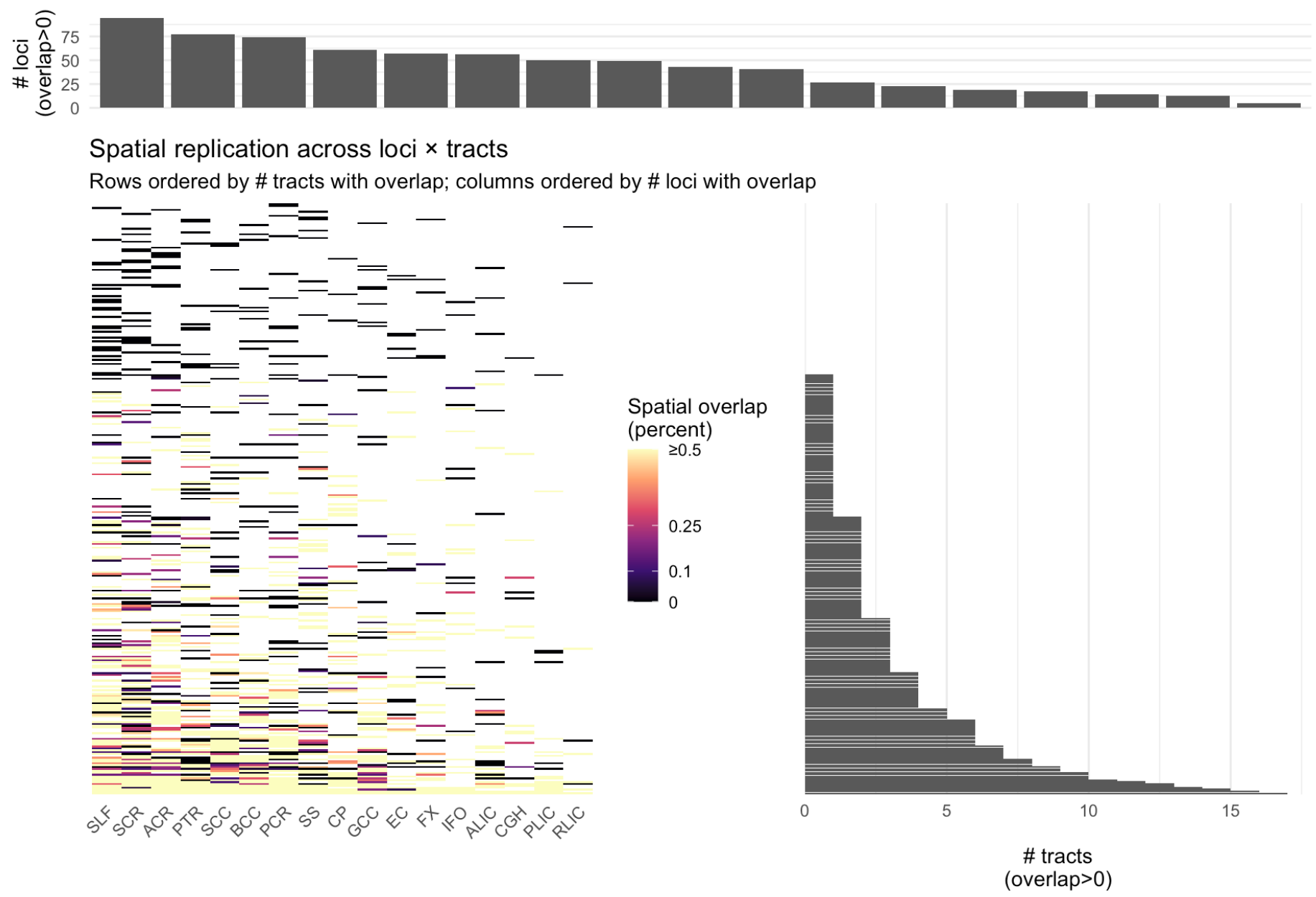

Fig. 13 Spatial replication of discovered loci across white-matter tracts. The heatmap shows the spatial overlap between discovery loci and replication signals across major white-matter tracts. Each row represents a locus and each column a tract; colors indicate the proportion of overlapping voxels between discovery and replication signals. Rows and columns are ordered by the number of tracts and loci showing overlap, respectively, with marginal bar plots summarizing these counts. Overall, 77% of the 315 discovered loci exhibited spatial overlap with replication signals, and 48% replicated with more than 50% spatial overlap, indicating substantial reproducibility of locus-specific white-matter association patterns.

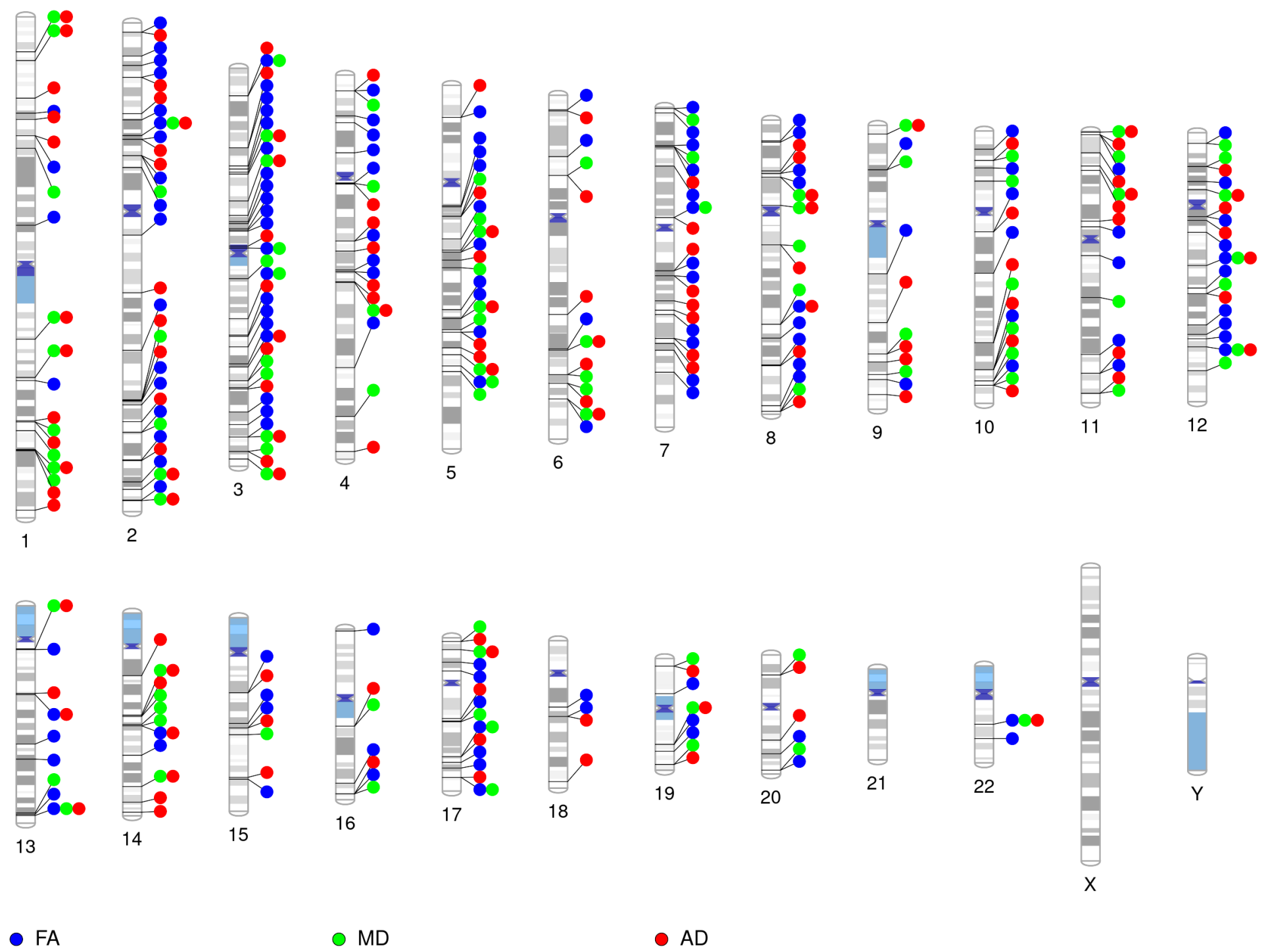

Fig. 14 Genomic distribution of loci associated with white-matter microstructure across diffusion MRI modalities. Chromosomal ideograms display the genomic locations of loci identified from voxel-wise GWAS of diffusion MRI measures. Points indicate lead variants for loci associated with each modality, including fractional anisotropy (FA; 315 loci), mean diffusivity (MD; 238 loci), and axial diffusivity (AD; 345 loci). Loci are distributed broadly across the genome, highlighting the polygenic architecture underlying variation in white-matter microstructure.

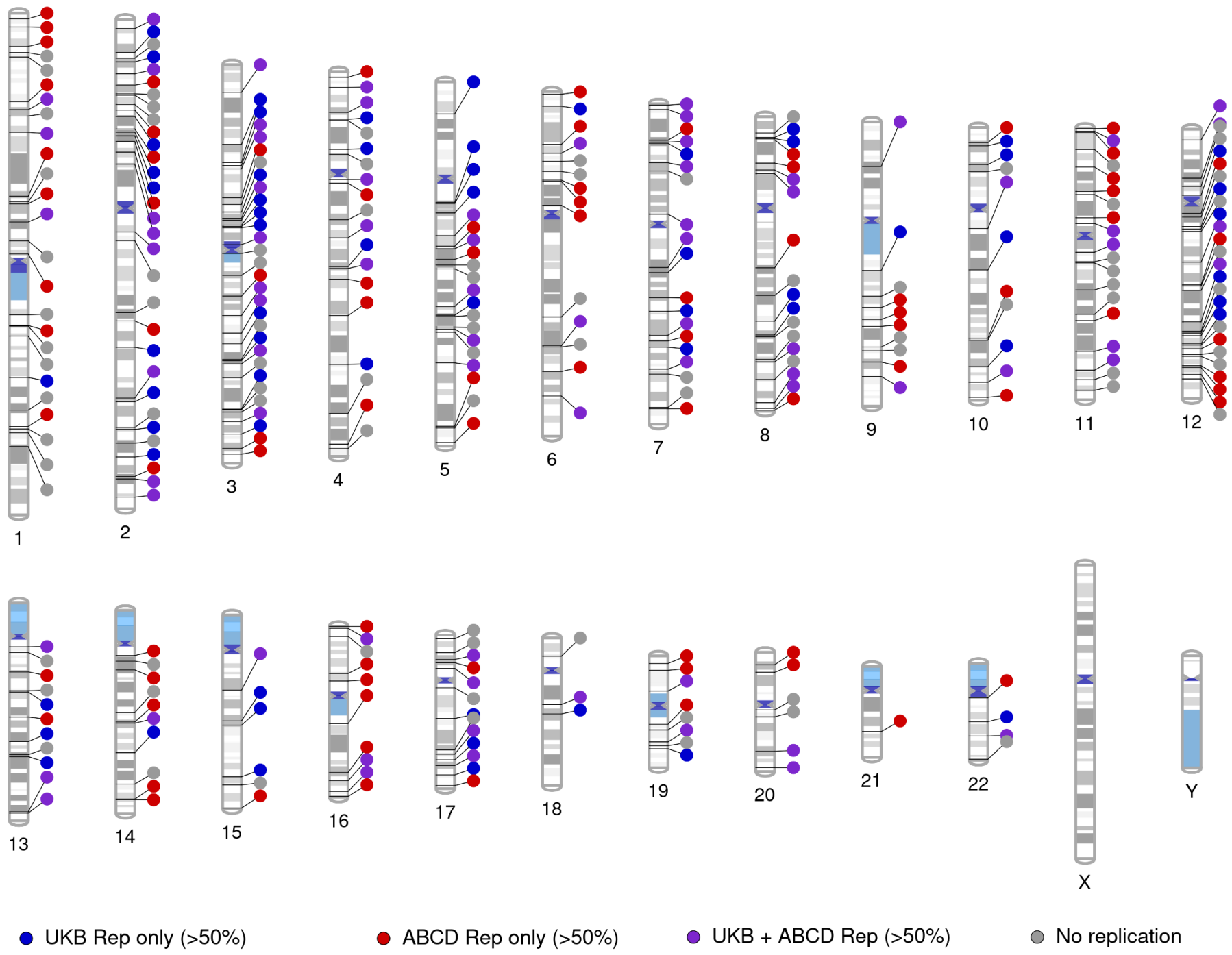

Fig. 15 Genomic distribution of FA loci across UKB and ABCD cohorts.
